# A non-parametric meta-analysis approach for combining independent microarray datasets: application using two microarray datasets pertaining to chronic allograft nephropathy

**DOI:** 10.1186/1471-2164-9-98

**Published:** 2008-02-26

**Authors:** Xiangrong Kong, Valeria Mas, Kellie J Archer

**Affiliations:** 1 Department of Biostatistics, Virginia Commonwealth University, Richmond, VA 23298, USA; 2 Department of Surgery, Virginia Commonwealth University, Richmond, VA 23298, USA; 3 Massey Cancer Center, Virginia Commonwealth University, Richmond, VA 23298, USA

## Abstract

**Background:**

With the popularity of DNA microarray technology, multiple groups of researchers have studied the gene expression of similar biological conditions. Different methods have been developed to integrate the results from various microarray studies, though most of them rely on distributional assumptions, such as the t-statistic based, mixed-effects model, or Bayesian model methods. However, often the sample size for each individual microarray experiment is small. Therefore, in this paper we present a non-parametric meta-analysis approach for combining data from independent microarray studies, and illustrate its application on two independent Affymetrix GeneChip studies that compared the gene expression of biopsies from kidney transplant recipients with chronic allograft nephropathy (CAN) to those with normal functioning allograft.

**Results:**

The simulation study comparing the non-parametric meta-analysis approach to a commonly used t-statistic based approach shows that the non-parametric approach has better sensitivity and specificity. For the application on the two CAN studies, we identified 309 distinct genes that expressed differently in CAN. By applying Fisher's exact test to identify enriched KEGG pathways among those genes called differentially expressed, we found 6 KEGG pathways to be over-represented among the identified genes. We used the expression measurements of the identified genes as predictors to predict the class labels for 6 additional biopsy samples, and the predicted results all conformed to their pathologist diagnosed class labels.

**Conclusion:**

We present a new approach for combining data from multiple independent microarray studies. This approach is non-parametric and does not rely on any distributional assumptions. The rationale behind the approach is logically intuitive and can be easily understood by researchers not having advanced training in statistics. Some of the identified genes and pathways have been reported to be relevant to renal diseases. Further study on the identified genes and pathways may lead to better understanding of CAN at the molecular level.

## Background

DNA microarray technology was launched in the early 90's. With its development and commercialization, it has been a popular tool for researchers to perform genome-wide analysis of gene expression profiles[1]. One major application of the technology is to compare, with simultaneous measurements on the expression of thousands of genes, the gene expression patterns under two or more different biological conditions, and to identify differentially expressed genes and their biological functions. One direct result of the popularity of the DNA microarray technology is the explosion of data generated from independent experiments that were designed to study similar biological conditions. Meta-analysis can thus be performed to integrate the results from these various DNA microarray experiments. Ordinarily, given a random sample, we assume that we can generalize the results and conclusions drawn from the sample to the population; however, the sample size for microarray experiments is usually small. By performing meta-analysis on data from multiple experiments, we can take advantage of the larger number of hybridized samples and make the findings more applicable to the full population.

Meta-analysis is the quantitative synthesis of a number of study results. A few groups of researchers have developed meta-analytic methods for combining results from multiple DNA microarray experiments [2-6]. The biological conditions to which such methods have been applied include prostate cancer [2,4,6], liver cancer [7], leukemia [8] breast cancer [3], pancreatic cancer [9], the common transcriptional profiles of neoplastic transformation and progression of multiple cancer types [5], and others [10].

A statistical challenge in performing a meta-analysis of microarray studies is that often samples are hybridized to different microarray platforms, and the technical differences among platforms lead to fundamental differences in the nature of the gene expression measurements produced. For example, the data for a custom spotted cDNA array are usually expressed as ratios of the intensity values corresponding to an experimental sample to the intensity values of a co-hybridized reference sample; while the data from an Affymetrix high density oligonucleotide microarray are absolute intensity values for the single channel. Therefore, data from different microarray platforms are not directly comparable, and it is essential to use the original values of gene expression from each platform to derive an "effect size" estimate that is independent of platform, thus rendering the different platforms comparable. The term "effect size" commonly used in meta-analysis refers to a standardized index measuring the effect associated with a treatment or covariate, or the magnitude of difference in gene expression in microarray studies [6]. Another challenge is how to integrate the effect size measurements from the individual studies.

To address these challenges, different effect size measurements and models for integrating microarray data have been proposed. In an early study [2], the authors performed a meta-analysis on two cDNA microarray studies and two Affymetrix oligonucleotide arrays, all of which compared the gene expression profiles between clinically localized prostate cancer and benign prostate tissue specimens. For each gene *g *(*g *= 1,⋯, *G*) and study *i *(*i *= 1,.., *I *= 4), they fit a simple linear regression model with tissue type as the covariate and expression measurement as the response. Then the ordinary least square estimate of the covariate coefficient divided by its standard error was used as the effect size measurement, which is equivalent to a t-statistic. Thus these gene-level effect size measurements are comparable among all the individual studies. To integrate these t-statistics across studies, a weighted average of t-statistics was calculated to obtain a global statistic for differential expression of each gene *g*, whereas the proportions of the sample sizes in study *i *to the overall sample size were used as the weights. To identify genes that were truly differentially expressed between the cancer and healthy tissues, the authors used permutation method by fitting linear models with permuted tissue labels to calculate the false discovery rate (FDR). Genes were declared differentially expressed corresponding to a specified FDR.

The same four prostate cancer microarray datasets were analyzed using an alternative method by Rhodes et al. [4]. They used the p-value *p*_*g*, *i *_calculated from the permutation t-test for each gene *g *in each study *i *to serve as the effect size measurement. To integrate across all the studies, Fisher's method for combining p-values, $S_{g}=-2\sum_{i=1}^{I}p_{g,i}$, was calculated for each gene. Under the null hypothesis that gene *g *did not have differential expression between the two groups, *S*_*g *_is chi-square distributed with degrees of freedom 2·*I*. Then the p-value for gene *g *based on the integral analysis of all the datasets can be calculated using the *χ*^2^_*df *= 2*I*_-distribution. Controlling the FDR at a certain level, differentially expressed genes could be identified as those with p-values less than a threshold determined by the FDR level.

Choi et al. [6] used a mixed-effects model approach to estimate the standardized mean expression difference for each gene *g *in each study *i*, and the study effect was treated as a random effect. A z-statistic *z*_*g*, *i*_, the effect size measurement, was calculated to be the ratio of the estimated mean expression differences to its standard error. To integrate across studies, the average z-statistic ${\bar{z}}_{g}=\frac{1}{I}\sum_{i=1}^{I}z_{g,i}$ was taken to be the summary z score for each gene. Then they used permutation technique to control the FDR and determine the cut-off value of the z scores. Genes with absolute z scores larger than the cut-off value were declared as significantly differentially expressed. These authors also incorporated a Bayesian method into the mixed-effects model to estimate the mean expression difference. They assumed different prior distributions for the standardized mean difference and the variance of the random study effect. The estimates of the effect size measurements were obtained from the corresponding posterior distributions.

Shen et al. [3] also used Bayesian framework to perform meta-analysis on microarray experiments studying breast cancer. However, the effect size measurement estimated using Bayesian hierarchical model was a self-defined probability: probability of expression, which was calculated based on a few distributional assumptions and ranged in [-1, 1]. After the probability of expression was obtained for each gene *g *in each study *i *(*i *= 1,.., *I*), they simply pooled the data from all *I *studies into one dataset and used univariate logistic regression technique to quantify genes relevance to breast cancer.

Nevertheless, to some degree, all these methods rely on the adherence of the data to a specified parametric distribution, such as the Gaussian distribution for the t-statistic methods and the mixed-effects model, or the different forms of prior distributions in the Bayesian context. However, commonly within each individual microarray study, only a small sample size is available, thus the normality assumption may not hold well. Further, it may be even more difficult to test the validity of the assumptions on the prior distributions and their parameters for Bayesian models. Some of the aforementioned methods are also somewhat computationally complicated and might be difficult to be understood by clinical researchers not having advanced training in statistics.

We obtained the data from two independent microarray experiments that compared the gene expression profiles between chronic allograft nephropathy (CAN) and normal functioning kidney allograft. Chronic Allograft Nephropathy is a major cause of graft loss and patient morbidity after kidney transplantation. The histopathology features of CAN are nonspecific and this makes it difficult to detect CAN before the occurrence of clinical manifestations. However, it is now well known that CAN may already be present in protocol biopsies before its clinical appearance [11]. It may be promising to characterize the gene expression pattern of transplant kidneys with CAN to use for prognosis of new kidney transplant recipients. A few studies used DNA microarray technology to compare, among patients who had kidney transplant, gene expression of biopsy samples from kidneys with CAN to those from the normal functioning kidneys. We obtained the gene expression data from two studies, which we refer to herein as the Hotchkiss study [12] and the Mas study [11]. Both used Affymetrix high density oligonucleotide microarrays to compare the gene expression profiles between CAN and normal functioning allograft, and identified lists of genes whose differential expression profiles could describe the molecular difference between CAN and the normal allograft. However, both of the studies suffered from the small sample size problem.

In this study, we sought to integrate the results from these two independent DNA microarray experiments. Herein we present a non-parametric approach for combining microarray data from various studies which does not suffer from the aforementioned limitations. This new method does not require any distributional assumption on the gene expression measurements, is logically intuitive, and is also easy to implement using statistical software. We will also report some of the biological findings from its application to integrate the two microarray studies.

## Results

The Hotchkiss study used Affymetrix HG-U133A human GeneChip arrays to measure the gene expression of 16 biopsies from patients with CAN and 6 biopsies from patients with normal functioning allograft [12]. The dataset was obtained upon request and the probe level data were already normalized and summarized using RMA (Robust Multichip Average) [13] method. The Mas study [11] was performed at Virginia Commonwealth University and the investigators used Affymetrix HG-U133A 2.0 human GeneChip arrays to measure the gene expression of 10 biopsies from patients with CAN and 4 with normal functioning allograft. For consistency, we also used RMA method to obtain probe set expression summaries from the original *CEL files of this study.

### Genes identified to be predictive of CAN

**Figure 1:**
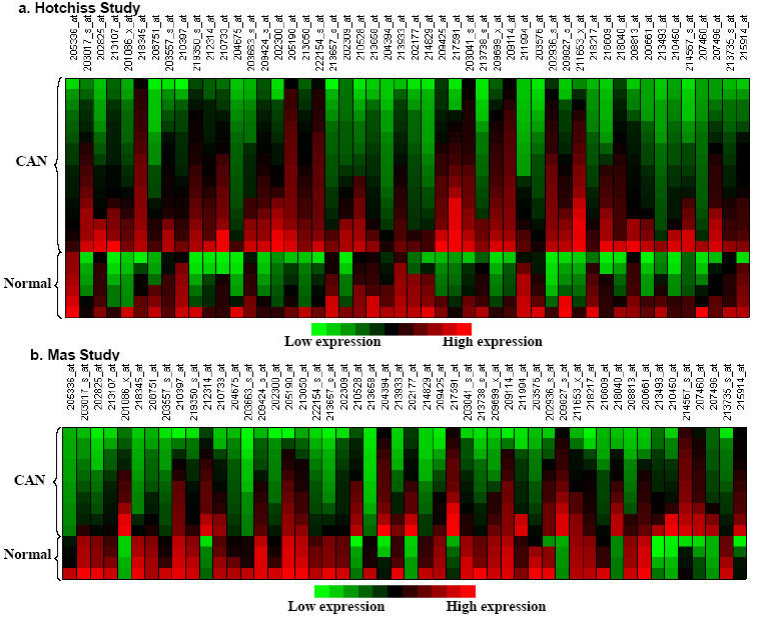
Heatmaps of the top 50 identified genes (a) Hotchkiss study. (b) Mas study.

Using our proposed meta-analysis method on the two microarray datasets, we identified 330 probe sets representing 309 genes that were significantly relevant to CAN. Associated with each gene was a score that measured this gene's ability to discriminate between CAN and normal allograft. The lower the score value, the better discriminative ability the gene had. The definition of the score and the procedure to obtain it are elaborated in the Methods section. Table 1 lists the first 10 genes that have the most desirable score values. Figure 1 is the heatmaps of the top 50 identified genes in the two studies. The complete list of the identified genes can be found in Additional File 1.

**Table 1:** 10 identified probe sets with the lowest ranked scores for discriminating CAN vs. Normal allograft

**LocusLink**	**AffyID**	**Gene Symbol**	**Unigene ID**	**Gene Name**	**Chromosome**	**Map**	**Pathway**	**Classification error rate (Score)**
5816	205336_at	PVALB	Hs.295449	parvalbumin	22	22q13.1	NA	0.01
117178	203017_s_at	SSX2IP	Hs.22587	synovial sarcoma, X breakpoint 2 interacting protein	1	1p22.3	Adherens junction	0.06
291	202825_at	SLC25A4	Hs.246506	solute carrier family 25 (mitochondrial carrier; adenine nucleotide translocator), member 4	4	4q35	Calcium signaling pathway	0.07
23043	213107_at	TNIK	Hs.34024	TRAF2 and NCK interacting kinase	3	3q26.2-q26.31	NA	0.09
6651	201086_x_at	SON	Hs.517262	SON DNA binding protein	21	21q22.11	NA	0.09
						21q22.1-q22.2		
55365	218345_at	HCA112	Hs.438823	NA	7	7q36.1	NA	0.09
8775	208751_at	NAPA	Hs.126938	N-ethylmaleimide-sensitive factor attachment protein, alpha	19	19q13.32	NA	0.09
5092	203557_s_at	PCBD1	Hs.3192	pterin-4 alpha-carbinolamine dehydratase/dimerization cofactor of hepatocyte nuclear factor 1 alpha (TCF1)	10	10q22	NA	0.1
1672	210397_at	DEFB1	Hs.32949	defensin, beta 1	8	8p23.2-p23.1	NA	0.1
56616	219350_s_at	DIABLO	Hs.169611	diablo homolog (Drosophila)	12	12q24.31	NA	0.1

**Figure 2:**
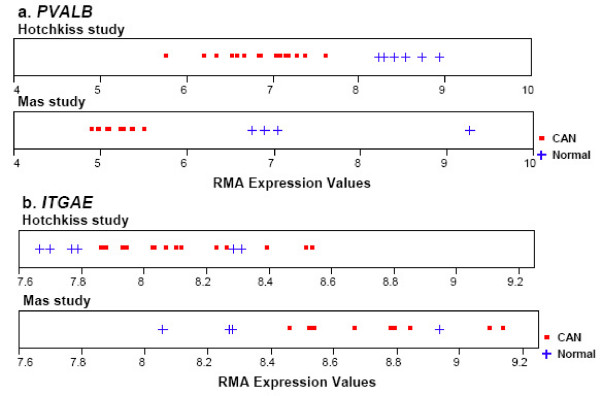
The differential expression pattern of gene (a) *PVALB *(Affy ID "205336_at") and gene (b) *ITGAE *(Affy ID "205055_at").

The gene *PVALB *(parvalbumin) has the most outstanding discriminative ability, or in other words, it has very distinct expression patterns between CAN and normal allograft, as shown in Figure 2a. Although the expression measurements in the two studies are of different ranges, 5.76–8.93 in the Hotchkiss study and 4.91–9.26 in the Mas study, the relative pattern is the same: the expression in CAN is lower than that in normal allograft. This gene encodes a high affinity calcium ion-binding protein and has been reported by multiple groups of researchers to be related to renal cell cancers (RCC) (eg: [14,15]). Wiesel et al. [16] found that "aggressive tumor growth of RCC requires close follow up in patients who received a renal allograft". The finding from this meta-analysis suggests that parvalbumin might be also relevant to the progression to CAN and deserves further study.

### Comparison between the genes identified by meta-analysis and SAM analysis in each individual study

The Significant Analysis of Microarray (SAM) method [17] was applied in both original studies by Hotchkiss and Mas to detect significantly differentially expressed genes at a false discovery rate (FDR) of 0.05. We applied SAM to the two datasets respectively. Controlling the FDR at 0.05, 26 probe sets are declared significant in Hotchkiss study, 10 of which are among the 330 probe sets identified by our meta-analysis; 2190 probe sets are declared significant in Mas study, 214 of which are among the 330 probe sets identified by our meta-analysis. Comparing the SAM analysis results of the two studies, only 2 probe sets are found to be in common.

The discordance among these results demonstrates that independent microarray experiments, even they use the same platform and study the same biological condition, may not be reproducible [18,19]. It also demonstrates the advantages of meta-analysis: since the overall sample size is larger than each individual study, it can find "small but consistent" [20] effect sizes which cannot be detected by analysis on individual studies. Also, some effect sizes might be significant in one study but not the other; meta-analysis can discard these inconsistent effect sizes which may be caused by the uniqueness of samples in that study.

A unique advantage of our meta-analysis method is that it can discern the differential gene expression patterns between CAN and normal allograft that cannot easily be described by some measurement of standardized mean difference in expression values, such as the t-statistic, which is the basis of the SAM method. An example is illustrated using the expression values of probe set "205055_at" in both studies. This is gene *ITGAE*, the fifty third in the identified gene list. In Figure 2b, plots of the distributions of *ITGAE *expression values in the two studies are presented. It is clear in both plots that the expression patterns are different between the two groups, with the expression of normal allograft samples generally being lower than that of CAN samples. However, because of the presence of several normal samples that have relatively high expression, the standardized means are not significantly different between the two groups. As in reality, even if a gene has been biologically validated to be up-regulated (or down-regulated) in a disease, relatively low (or high) expression may still be observed in a few samples. The two-sided t-test for *ITGAE *in the Hotchkiss study yields a p-value 0.20, and 0.14 in the Mas study. Therefore, models based on t-statistic will not recognize this gene as having differential patterns between the two groups, although the expression patterns are indeed different enough to differentiate the samples.

### Comparison between the genes identified by our non-parametric meta-analysis and a t-statistic based meta-analysis

We also performed a meta-analysis on the two CAN studies using a commonly used parametric approach that is based on t-statistic, and compared the result to that from our non-parametric meta-analysis approach. The t-statistic based method is derived from [4] and is described as in the Background section. To control for the FDR, Benjamini and Yekutieli's method [21] of adjusting the p-values was used. At an FDR level of *α *= 0.01, 2026 probe sets are identified to have significantly differential expression between the normal allograft and CAN samples. The majority of the 330 probe sets identified by our non-parametric meta-analysis are also identified by this parametric meta-analysis; however 73 out of the 330 probe sets are not identified by the parametric analysis, including the above mentioned probe set ''205055_at'', which has an adjusted p-value 0.41. Most of the 73 probe sets indeed have the similar differential expression pattern as seen in Figure 2b, which cannot be depicted by the t-statistic based model, yet is a reflection of what might be observed in reality.

### Biological pathways associated with the identified genes

The meta-analysis was performed on the individual gene level and assigned a score for each gene that quantified its difference in expression between CAN and normal allograft. However, genes do not express independently, especially when they are components of the same biological pathway. Therefore, we also examined the identified genes at the pathway level.

Among the 330 identified genes using our non-parametric approach, 129 have been annotated in KEGG (Kyoto Encyclopedia of Genes and Genomes) pathway database, and they are components of 114 pathways. For each pathway we used Fisher's exact test with *α *= 0.01 to test the null hypothesis of no difference against the alternative that significantly more genes were represented in the list of the identified genes of that pathway than would be expected by chance. Six pathways were found to be significantly over-represented. Table 2 lists the pathway names and the number of identified genes in the pathways.

**Table 2:** Over-represented KEGG pathways by Fisher exact test, at significance level 0.01

**Pathway Name**	**No. of Identified Probe Sets Relevant to CAN in the Pathway**	**Total No. of Probe Sets in the Pathway**	**Fisher Exact Test p-value**
Oxidative phosphorylation	14	158	0
ATP synthesis	7	59	0
Citrate cycle (TCA cycle)	5	42	0
Reductive carboxylate cycle (CO2 fixation)	3	15	0
Cholera – Infection	6	84	0.01
Methionine metabolism	3	23	0.01

As an illustration, we examined the most significantly over-represented pathway in the literature. Oxidative phosphorylation is a process of cellular respiration and consisted of five complexes located in the inner mitochondrial membrane. ATP synthesis is the final protein complex in the metabolic pathway. It has been reported that mitochondrial disorders can sometimes give rise to kidney dysfunction [22]. The finding from this meta-analysis indicates that the abnormal activities in the process of oxidative phosphorylation might be related to the development of interstitial fibrosis and tubular atrophy, which are specific features of CAN.

### Allograft status prediction on 6 unpublished samples

Predictions were performed on 6 additional transplant kidney biopsies that were procured and hybridized to Affymetrix HG-U133A 2.0 GeneChip arrays from Mas et al. after their previous study. These 6 samples were all diagnosed as CAN by experienced pathologists based on the histological observations. Each identified gene was used as the single predictor to predict the class labels of the 6 samples, using classifiers derived from the Hotchkiss data and Mas data respectively. The weighted average of the error rates associated with each identified gene is recorded in the last column of Additional File 1, where the weight corresponding to each study was taken to be the proportion of its sample size in the total combined sample size.

When using the classifiers derived from Hotchkiss data and Mas data respectively with all the 330 identified genes expression measurements as predictors, the predicted classes all conformed to their true diagnostic classes.

### Simulation Study

We conducted a simulation study to investigate the generalizability of our non-parametric meta-analysis approach. Two datasets were generated independently to simulate the RMA expression summary data observed from the two Affymetrix GeneChip experiments in which CAN was studied. We simulated expression values for *G *= 2000 genes in each of two studies, with a percent *p *= 10% of genes to be truly differentially expressed between the disease group and normal group. The sample sizes were *n*_1, *d *_= 4 or 8, *n*_1, *norm *_= 6 or 10 in study I for the disease and normal groups respectively; similarly, the samples sizes were *n*_2, *d *_= 6 or 10, *n*_2, *norm *_= 15 or 15 in study II for the two groups respectively. These small samples sizes were used to mimic the CAN meta-analysis. To identify differentially expressed genes, the non-parametric meta-analysis approach and permutation t-test based approach were then applied on the simulated datasets. We note that because of the small sample sizes in the disease group, mixture-effects model based meta-analysis approach is not likely to be appropriate to use. The performance of the applied two approaches was evaluated by two statistics: sensitivity (percentage of correctly identified differentially expressed genes in the pool of truly differentially expressed genes); and specificity (percentage of identified non-differentially expressed genes in the pool of truly non-differentially expressed genes).

#### Simulation model and algorithm

The intensity of a gene was generated using the following model:

*y*_*g*, *i*, *j *_= *μ*_*g *_+ *I*(*i *= 2)·*β*_(*i*)*g *_+ *I*(*j *= 2)·*γ*_(*j*)*g *_+ *ε*_*i*(*j*)*g*_

where *y*_*g*, *i*, *j *_is the intensity for gene *g*(*g *= 1,..., *G *= 2000) in study *i *= 1, 2 and group *j *= 1 (normal) and 2 (disease); *I*() is the indicator function; *μ*_*g *_is the overall gene *g *effect; *β*_(*i*)*g *_is the study effect; *γ*_(*j*)*g *_is the group effect; and *ε*_*i*(*j*)*g *_is the random error term nested within group, allowing different variability in different groups. The procedure used in the generation of the datasets is outlined in the following steps:

1). Generate the gene effects vector *μ *= (*μ*_1_,..., *μ*_*G*_)' ~ multivariate *N*(*μ*_0_, Σ), where *μ*_0 _= (*μ*_0_, *μ*_0_,..., *μ*_0_), where *μ*_0_~Uniform (4.5,9) with probability 0.9 and Uniform(6,12) with probability 0.1; and Σ~[(*n*_1 _- 1)·Σ_1 _+ (*n*_2 _- 1)·Σ_2_]/(*n*_1 _+ *n*_2 _- 2), where Σ_1 _and Σ_1 _are the sample variance-covariance matrices of 2000 randomly selected genes in the Hotchkiss study and Mas study, respectively. Thus the correlation structure in the genes is preserved in the simulated data.

2). For samples in Study II, generate the study effect for each gene: *β*_(*i*)*g*_~*N*(*μ*_*β*_, *σ*_*β*_^2^), where *μ*_*β*_~Uniform (0,2) and *σ*_*β*_^2^~Uniform(0,0.5).

3). Randomly select *p *= 10% of the genes to be truly differentially expressed genes. Additionally randomly select some samples to represent the diseased group. Generate the group effect for each of these genes in these disease samples: *γ*_(*j*)*g*_~*N*(*μ*_*γ*_, *σ*_*γ*_^2^), where *μ*_*γ*_~Uniform(0.2,1) with probability 0.4, Uniform(1, 2.2) with probability 0.1, Uniform(-1,-0.2) with probability 0.4, and Uniform(-2.3,-1) with probability 0.1; *σ*_*γ*_^2^~Uniform(0.4,0.5).

4). Generate the random error for each gene in each sample: *ε*_*i*(*j*)*g*_~*N*(0,0.1).

#### Simulation result

We ran 30 simulations and the means and standard deviations (SD) of the sensitivity and specificity statistics using the two approaches are reported in Table 3. The small SDs indicate that both our KNN based non-parametric approach and the t-statistic based parametric approach have stable performance over the 30 simulation runs. Using either approach, the sensitivity is increased in scenario II where a larger sample size is available compared to scenario I. Under both scenarios, our approach outperforms the t-statistic based approach, either in terms of sensitivity or specificity. The specificity statistic is always high (>95%), as is expected since 90% of the *G *= 2000 genes truly have non-differential expression.

**Table 3:** Mean sensitivity and specificity using the non-parametric approach and t-statistic based approach from the 30 simulations, under two scenarios of different sample sizes. (SD: standard deviation)

Meta-analysis approach	Scenario I: *n*_1, *d *_= 4, *n*_1, *norm *_= 6 *n*_2, *d *_= 6, *n*_2, *norm *_= 15		Scenario II: *n*_1, *d *_= 8, *n*_1, *norm *_= 10 *n*_2, *d *_= 10, *n*_2, *norm *_= 15	
	
	Sensitivity (%) (SD)	Specificity (%) (SD)	Sensitivity (%) (SD)	Specificity (%) (SD)
KNN based non-parametric	78.15 (2.90)	99.83 (0.10)	84.83 (2.68)	99.96 (0.06)
t-statistic based parametric	75.77 (3.19)	96.02 (0.78)	77.00 (3.01)	96.00 (0.55)

## Discussion

The two microarray experiments analyzed in this meta-analysis were both performed using Affymetrix platform, whereas the Hotchkiss study utilized the HG-U133A arrays and Mas et al. used HG-U133A 2.0 (version 2) arrays. Other than some differences in the control probe sets, the probe set IDs are identical between the two versions of HG-U133A arrays. Therefore, gene mapping between datasets was not a challenging step.

Hu et al. [23] developed a gene hybridization quality measure for Affymetrix DNA microarray platform and incorporated it as a quality weighing strategy into the effect size estimation in Choi et al.'s mixture-effect model. We also considered using a hybridization quality measurement as a weighing system when deriving for each gene the score that described the gene's ability in discerning CAN vs. normal allograft. However, the dataset obtained from the Hotchkiss study is already probe set level data summarized by RMA method; therefore, we were unable to implement a quality measure in the analysis.

The meta-analysis method we proposed is applicable to situations where multiple microarry platforms are involved. However, if the data are from the same platform, the same normalization method should be used. The Tumor Analysis Best Practices Working Group compared different probe set expression summary algorithms for Affymetrix GeneChip arrays and claimed "different probe set interpretation algorithms lead to different results" [24]. They often observed only "~50% concordance in general data output in their own work between comparisons of two different algorithms". Therefore, a good expression summary algorithm is essential for performing down-stream analysis. Shippy et al. [25] used RNA sample titrations to assess microarray platform performance and normalization techniques [26-30]. It is not the research focus in this paper, and we suggest applying the same algorithm on datasets from the same platform.

It is well known that genes, especially genes in a common pathway, are correlated. We considered starting the meta-analysis from a pathway level, i.e. first identifying pathways that might be relevant to the progress of CAN; and then focusing on the individual genes in those pathways and finding out genes whose expression patterns were differential between CAN and normal allograft. However, since only less than 30% of the genes measured on the Affymetrix HG-U133A chips have been annotated with known KEGG pathway information, we decided to perform the analysis at the gene level. This may help researchers understand gene functions that are still unknown and avoid throwing away 70% of the available data.

Chronic allograft nephropathy is a complex entity at both histological and molecular level. We identified 330 sequences whose differential expression patterns could distinguish between CAN and normal allograft. The functions of most of the identified genes are not well understood yet. More studies on these genes, especially on those at the top of the list, may lead to a better understanding of the progression of CAN at the molecular level. Furthermore, each gene is associated with a score that measures its degree of differential expression between the two groups. All the identified genes have scores below the pre-determined threshold 0.1737. By adjusting the threshold based on prior expertise knowledge about CAN, more or less genes can be identified for further study. To utilize a smaller set of genes for prognosis on kidney transplant recipients, the genes with the lowest score values can be selected, such as the 10 listed in Table 1. Further study on the expression of these identified genes in the kidney transplant recipients might be very informative in terms of prognosticating the development of CAN.

## Conclusion

In this paper, we present a new meta-analysis technique for combining DNA microarray studies by analyzing two independent microarray studies comparing the gene expression of CAN and normal allograft. This is a non-parametric approach that is statistically easy to understand, and can discern differential expression pattern that may not be detected by t-statistic based models and mixture-effects model. Although the new method is applied to combine two microarray studies of the same platform, its' use is by no means limited to a single platform and can be used to different platforms without difficulty.

## Methods

The probe level data from the two independent Affymetrix microarray studies were normalized and summarized using RMA method. To assess the sample quality, we calculated the 3' :5' ratios for three Affymetrix control probe sets corresponding to human genes *GAPDH, ISGF *and *β*-*actin*. For both studies, all the ratios were less than 3, the threshold recommended by Affymetrix. Therefore, sample degradation did not seem to be a problem in both studies, and thus we regarded all samples as useful. Before combining the data across the two independent studies, all Affymetrix control probe sets were excluded, leaving 22,215 probe sets that were common to both studies.

### Definition and calculation of effect size measurement in individual studies

We considered finding in each individual study for each gene an effect size measure that could quantify the gene's ability in discriminating CAN from normal allograft. This was essentially determined by the degree of difference in the gene's expression between the two groups, and reflected as how well its expression measurements could classify the samples. Conceptually, if a gene is irrelevant to CAN, using its expression to determine class membership would appear as random guessing. On the other hand, if a gene is an important predictor in distinguishing class, i.e. it has different expression patterns between CAN and normal allograft, we will expect to correctly classify most of the samples using its expression, and the misclassification error rate will be close to 0.

Therefore, within study *i *(*i *= 1, 2) we used the expression for each gene *g *(*g *= 1,..., 22215) as the single predictor variable and applied the K-Nearest-Neighbor (KNN) classification method to develop a classifier for the *n*_*i *_samples in study *i*. Thereafter, the KNN classifier was used to predict class label for each sample. The predicted labels were compared with their corresponding true class labels and an unbiased estimate of the misclassification error rate, denoted as *err*_*g*, *i*_, was calculated. This error rate estimate measured this gene's discriminative ability and is defined as our effect size statistic.

Using KNN, we estimate directly at each observation the posterior probability of each class, given the observed predictor (gene expression), as the proportion of that class among the k nearest "neighbors" of the target observation. Then the classification for the target observation is the class which had the largest estimated posterior probability. The advantages of KNN include that it does not require any distributional assumptions, and it has reasonable performance comparing to other classification methods. A property of the large-sample behavior of KNN is described in the following theorem [31]:

THEOREM: Let E* denote the error rate of the Bayes rule in a *C*-class problem, i.e. the best possible error rate for the classification problem. Then the error rate of KNN converges in *L*_1 _as the size of the training set increases to a value *E*_1 _bounded below by E* and above by $E^{*}\times (2-\frac{C}{C-1}E^{*})$.

For a two-class problem, E* = *E*(min(*P*(class I|*x*), *P*(class II|*x*))) ≤ 0.5, where *x *is the vector of predictors. Thus the asymptotic upper bound of the error rate of KNN is E* × (2 - 2E*) ≤ 0.5, which means that KNN has asymptotic performance as good as the performance of the Bayes rule. When the sample size is small, as is often the case in microarray studies, KNN is also suitable to use as Ripley [32] indicates that most other non-parametric classification methods, such as kernel density estimation based methods, aim to model the class-conditional densities and thus need a very large training set to be successful. Therefore, due to the non-parametric nature of directly modeling the posterior probabilities and the good performance of KNN, it is applied to appropriately quantify the true discriminative ability of each gene.

We used an odd value of *k *(the number of neighbors) to avoid ties, and chose *k *= 3 considering in the Mas study, only 4 samples were available in the normal allograft group. For microarray studies with larger sample sizes, *k *can be determined using cross-validation and can be different for the individual studies. Since the data from both studies were summarized using RMA method and thus the scales of the data are similar, the Euclidean distance was used to quantify the similarity in the predictor gene's expression profile between samples and determine the ''nearest neighbors'' for each sample.

The apparent misclassification error rate, which is the number of misclassified observations in the training dataset divided by the total number of samples in the training dataset, tends to under-estimate the true misclassification error rate [32]. Therefore, we used the refined bootstrap estimate to obtain an unbiased estimate for the misclassification error rate [33]. The refined bootstrap estimate corrects the apparent error rate estimate by adding the optimism due to estimating the error rate using the same observations that are also used in deriving the classifier. For each gene *g *in each individual study *i*, we generated *B *= 100 bootstrap resamples. For each bootstrap resample, we used the bootstrap sampled gene's expression measurements as predictor values and applied the KNN to develop the classifier. The classifier was used to predict the class labels for the bootstrap samples, as well as the original samples, respectively. If *R*_*boot*,(*b*), *g*, *i *_denotes the misclassification error rate in the *b*^*th *^bootstrap samples, and *R*_*ori*,(*b*), *g*, *i *_denotes the misclassification rate in the original samples using the classifier built from the *b*^*th *^bootstrap samples; then $R_{opt,g,i}=\frac{1}{B}\sum_{b=1}^{B}\left(R_{ori,(b),g,i}-R_{boot,(b),g,i}\right)$ is the optimism estimate. Therefore, the unbiased estimate of misclassification error rate *err*_*g*, *i *_is the apparent error rate in the original samples, which uses the classifier built from the original samples themselves, plus the optimism estimate from bootstrap samplings, i.e., *err*_*g*, *i *_= *R*_*g*, *i *_+ *R*_*opt*, *g*, *i*_, where *R*_*g*, *i *_denotes the apparent error rate and *g *= 1, 2,..., 22215; *i *= 1, 2.

It is noteworthy to notice that this step can be carried out on microarray datasets from any platforms. Although different platforms may yield distinct scales of numerical measurements of gene expression, as long as there exists a relative different expression pattern between the two classes, the discrimination method can be applied to quantify each gene's association with the class label.

### Integration of the effect sizes across studies

After we obtained the study-specific effect size measurements (*err*_*g*, *i*_) for each gene *g*, we calculated the weighted average across studies as the combined effect size (or score) of this gene. The weight corresponding to each study was taken to be the proportion of its sample size in the total combined sample size. The combined effect size for gene *g *across studies is $e\bar{r}r_{g.}=\sum_{i=1}^{2}\frac{n_{i}}{\sum_{i=1}^{2}n_{i}}err_{g,i}$.

### Identification of genes relevant to CAN

**Figure 3:**
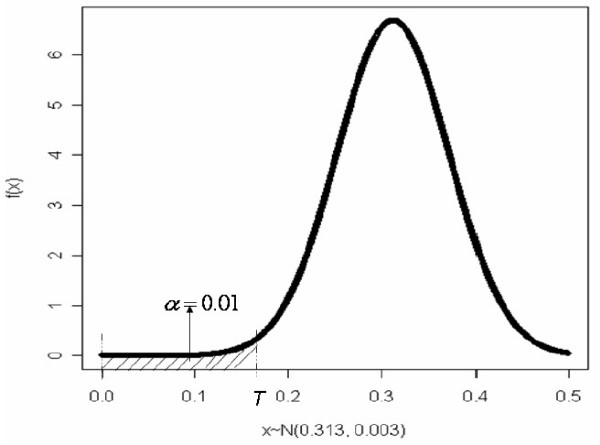
Normal density plot to illustrate the selection of the threshold for the scores.

To identify genes capable of distinguishing between CAN and normal allograft, we wanted to identify the $e\bar{r}r_{g.}$ estimates that are "equivalent" to 0. To do so, we determined a threshold *T *such that if the probability of a score being less than the threshold was less than *α *= 0.01, the score was considered to be equivalent to 0, i.e., *P*($e\bar{r}r_{g.}$ - 0 <*T *| *g*) ≤ *α*. The Q-Q plot of $e\bar{r}r_{g.}$ for the 22,215 probe sets (not shown here) demonstrates that $e\bar{r}r_{g.}$ are approximately normally distributed. Therefore, assuming $e\bar{r}r_{g.}$ ~ *N*(*μ*, *σ*^2^), the threshold *T *is the quantile such that *P*(*x *- 0 <*T*|*x *~*N*(*μ*, *σ*^2^)) = *α*, and estimating T by plugging in the moment-based estimates for *μ *and *σ*^2^. This is illustrated graphically in Figure 3. The genes whose scores are less than the threshold are identified as being relevant to CAN. The analyst can adjust *α *to identify either a larger or smaller number of genes.

**Figure 4:**
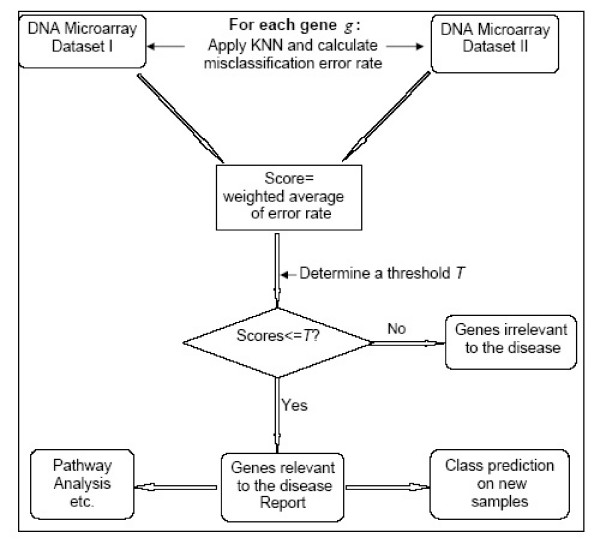
Flowchart illustrating our non-parametric meta-analysis approach.

The entire meta-analysis method is summarized in Figure 4.

The analysis was conducted in the R 2.4.0 environment on a PC with Intel Core Duo CPU@2.0G × 2 and 2.0G RAM. The average calculation time needed for each gene is about 0.9 second. More efficient and faster performance of the algorithm can be realized when the implementation in the C language is available. Our R code for analyzing the two CAN studies can be found in Additional File 2.

### Identification of over-represented KEGG pathways

To identify the over-represented pathways associated with the identified genes, we first filtered the whole gene list by excluding probe sets that had not been annotated in KEGG pathway database, and denoted the number of remained probe sets as *G*_0_. Among the remaining probe sets, we let *G*_*ID *_denote the number of probe sets that were identified to be relevant to CAN, and let *G*_0, *p *_denote the number of probe sets that were components of a pathway *p*(*p *= 1, 2,..., *P*). Among the *G*_*ID *_identified probe sets, *G*_*ID*, *p *_probe sets were in the pathway *p*. Then we performed a Fisher's exact test [34] on the 2 × 2 table as in Table 4 to test whether this pathway *p *was over-represented in the identified genes at a significant level 0.01.

**Table 4:** 2 × 2 Table for testing whether pathway *j *was over-represented in the identified genes (To test *H*_0_: the number of genes in pathway *p *is independent of the number of identified genes relevant to CAN. Vs. *H*_1_: the number of genes in pathway *p *is over-represented in the identified genes relevant to CAN, since all the marginal values are given, Fisher's exact test is used. The p-value from the test is $P(G_{ID,p}\geq g_{ID,p})=\sum_{t\geq g_{ID,p}}\frac{\left(\begin{array}{c} G_{ID}\\ t \end{array}\right)\left(\begin{array}{c} G_{0}-G_{ID}\\ G_{0,p}-t \end{array}\right)}{\left(\begin{array}{c} G_{0}\\ G_{0,p} \end{array}\right)}$, where *g*_*ID*, *p*_denotes the observed value of *G*_*ID*, *p*_)

	**No. of Genes in Pathway *p***	**No. of Genes Not in Pathway *p***	**Total**
**No. of Identified Genes Relevant to CAN**	*G*_*ID*, *p*_	*G*_*ID *_- *G*_*ID*, *p*_	*G*_*ID*_
**No. of Genes Irrelevant to CAN**	*G*_0, *p *_- *G*_*ID*, *p*_	*G*_0 _- G_0, *p *_- (*G*_*ID *_- *G*_*ID*, *p*_)	*G*_0 _- G_*ID*_
**Total**	*G*_0, *p*_	*G*_0 _- G_0, *p*_	*G*_0_

### Prediction on 6 unpublished samples

To predict the class labels of the 6 additional samples, we first normalized and summarized the *.CEL files using RMA method. It was noticed that although a gene's differential expression pattern might be similar in both studies, the numerical values between the studies took on different ranges. As can be seen in Figure 1, the gene *PVALB *was significantly differentially expressed between CAN and normal allograft in both studies; however, the measurements on CAN samples in the Hotchkiss study were in the range of (5.76, 7.61), while in the Mas study they were in the range of (4.90,5.50). Therefore, a global shift on the RMA normalized measurements existed between the two independent studies. Because the six unpublished samples may have been processed by different technicians, the shift on the measurements may also be present between these samples and the earlier two studies.

In order to diminish the impact of the global shift and make the classifier derived from the previous two studies applicable to these unpublished samples, we centered the measurements of each identified gene by subtracting its median in each individual dataset. Next the KNN algorithm was run to build classifiers respectively with the centered data in the Hotchkiss study and Mas study. Then the two classifiers were applied respectively to classify the 6 samples and the predicted results were compared to their true classes.

## Authors' contributions

XK developed the method, performed the statistical analysis, wrote programming code, and drafted the manuscript. VM provided data, contributed to the scientific understanding of the clinical implications of CAN in renal transplant recipients, and contributed to revising the manuscript. KJA conceived the study and provided extensive and detailed comments on the statistical analysis and on the manuscript. All authors read and approved the final manuscript.

## Supplementary Material

Additional file 1 The complete list of the identified 330 probe sets that were significantly relevant to chronic allograft nephropathy. Click here for file

Additional file 2 The R code to perform our non-parametric meta-analysis on the two CAN microarray studies. Click here for file
