# Supplementary material for: A non-parametric meta-analysis approach for combining independent microarray datasets: application using two microarray datasets pertaining to chronic allograft nephropathy
Source: BMC Genomics. 2008 Feb 26;9:98. doi: 10.1186/1471-2164-9-98 (PMC2276496; doi:10.1186/1471-2164-9-98)
Supplement: Additional file 1 — The complete list of the identified 330 probe sets that were significantly relevant to chronic allograft nephropathy. [file 1471-2164-9-98-S1.html]

Differentially Expressed Genes


| Differentially Expressed Genes | | | | | | | | | |
|  |  | | | | | | | | |
| Non-parametric Meta-analysis identified genes | | | | | | | | | |
|  |  | | | | | | | | |
| LocusLink | AffyID | Gene Symbol | UnigeneID | Gene Name | Chromosome | Map | Pathway | Score | Weighted Average of Error Rates on 6 new samples |
| 5816 | 205336\_at | PVALB | Hs.295449 | parvalbumin | 22 | 22q12-q13.1 | NA | 0.01 | 0.17 |
| 22q13.1 |
| 117178 | 203017\_s\_at | SSX2IP | Hs.22587 | synovial sarcoma, X breakpoint 2 interacting protein | 1 | 1p22.3 | Adherens junction | 0.06 | 0.5 |
| 291 | 202825\_at | SLC25A4 | Hs.246506 | solute carrier family 25 (mitochondrial carrier; adenine nucleotide translocator), member 4 | 4 | 4q35 | Calcium signaling pathway | 0.07 | 0.37 |
| 23043 | 213107\_at | TNIK | Hs.34024 | TRAF2 and NCK interacting kinase | 3 | 3q26.2-q26.31 | NA | 0.09 | 0.33 |
| 6651 | 201086\_x\_at | SON | Hs.517262 | SON DNA binding protein | 21 | 21q22.1-q22.2 | NA | 0.09 | 0.27 |
| 21q22.11 |
| 55365 | 218345\_at | HCA112 | Hs.438823 | NA | 7 | 7q36.1 | NA | 0.09 | 0.06 |
| 8775 | 208751\_at | NAPA | Hs.126938 | N-ethylmaleimide-sensitive factor attachment protein, alpha | 19 | 19q13.32 | NA | 0.09 | 0 |
| 5092 | 203557\_s\_at | PCBD1 | Hs.3192 | pterin-4 alpha-carbinolamine dehydratase/dimerization cofactor of hepatocyte nuclear factor 1 alpha (TCF1) | 10 | 10q22 | NA | 0.1 | 0.19 |
| 1672 | 210397\_at | DEFB1 | Hs.32949 | defensin, beta 1 | 8 | 8p23.2-p23.1 | NA | 0.1 | 0.33 |
| 56616 | 219350\_s\_at | DIABLO | Hs.169611 | diablo homolog (Drosophila) | 12 | 12q24.31 | NA | 0.1 | 0.33 |
| 23231 | 212314\_at | KIAA0746 | Hs.479384 | NA | 4 | 4p15.2 | NA | 0.1 | 0.17 |
| 23471 | 210733\_at | TRAM1 | Hs.491988 | translocation associated membrane protein 1 | 8 | 8q13.3 | NA | 0.1 | 0.3 |
| 6715 | 204675\_at | SRD5A1 | Hs.552 | steroid-5-alpha-reductase, alpha polypeptide 1 (3-oxo-5 alpha-steroid delta 4-dehydrogenase alpha 1) | 5 | 5p15 | Bile acid biosynthesis | 0.1 | 0.37 |
| Androgen and estrogen metabolism |
| 9377 | 203663\_s\_at | COX5A | Hs.401903 | cytochrome c oxidase subunit Va | 15 | 15q25 | Oxidative phosphorylation | 0.1 | 0.2 |
| 23600 | 209424\_s\_at | AMACR | Hs.508343 | alpha-methylacyl-CoA racemase | 5 | 5p13.2-q11.1 | NA | 0.1 | 0.33 |
| 10542 | 202300\_at | HBXIP | Hs.439815 | hepatitis B virus x interacting protein | 1 | 1p13.3 | NA | 0.11 | 0.19 |
| 5357 | 205190\_at | PLS1 | Hs.203637 | plastin 1 (I isoform) | 3 | 3q23 | NA | 0.11 | 0.5 |
| 23242 | 213050\_at | COBL | Hs.99141 | cordon-bleu homolog (mouse) | 7 | 7p12.1 | NA | 0.11 | 0.44 |
| 26010 | 222154\_s\_at | DNAPTP6 | Hs.120323 | NA | 2 | 2q33.1 | NA | 0.11 | 0.13 |
| 374655 | 213657\_s\_at | ZNF710 | Hs.459311 | zinc finger protein 710 | 15 | 15q26.1 | NA | 0.11 | 0.33 |
| 4522 | 202309\_at | MTHFD1 | Hs.632340 | methylenetetrahydrofolate dehydrogenase (NADP+ dependent) 1, methenyltetrahydrofolate cyclohydrolase, formyltetrahydrofolate synthetase | 14 | 14q24 | Glyoxylate and dicarboxylate metabolism | 0.11 | 0.37 |
| One carbon pool by folate |
| 3140 | 210528\_at | MR1 | Hs.101840 | major histocompatibility complex, class I-related | 1 | 1q25.3 | NA | 0.11 | 0.27 |
| 374655 | 213658\_at | ZNF710 | Hs.459311 | zinc finger protein 710 | 15 | 15q26.1 | NA | 0.11 | 0.17 |
| 8501 | 204394\_at | SLC43A1 | Hs.591952 | solute carrier family 43, member 1 | 11 | 11p11.2-p11.1 | NA | 0.11 | 0.27 |
| 5733 | 213933\_at | PTGER3 | Hs.445000 | prostaglandin E receptor 3 (subtype EP3) | 1 | 1p31.2 | Calcium signaling pathway | 0.11 | 0.27 |
| Neuroactive ligand-receptor interaction |
| 2621 | 202177\_at | GAS6 | Hs.369201 | growth arrest-specific 6 | 13 | 13q34 | NA | 0.11 | 0.33 |
| 10157 | 214829\_at | AASS | Hs.156738 | aminoadipate-semialdehyde synthase | 7 | 7q31.3 | Lysine biosynthesis | 0.11 | 0.33 |
| Lysine degradation |
| 23600 | 209425\_at | AMACR | Hs.508343 | alpha-methylacyl-CoA racemase | 5 | 5p13.2-q11.1 | NA | 0.11 | 0.5 |
| 6498 | 217591\_at | SKIL | Hs.581632 | SKI-like | 3 | 3q26 | NA | 0.11 | 0.1 |
| 3920 | 203041\_s\_at | LAMP2 | Hs.496684 | lysosomal-associated membrane protein 2 | X | Xq24 | NA | 0.11 | 0 |
| 498 | 213738\_s\_at | ATP5A1 | Hs.298280 | ATP synthase, H+ transporting, mitochondrial F1 complex, alpha subunit 1, cardiac muscle | 18 | 18q12-q21 | Oxidative phosphorylation | 0.11 | 0.44 |
| ATP synthesis |
| 1646 | 209699\_x\_at | AKR1C2 | Hs.460260 | aldo-keto reductase family 1, member C2 (dihydrodiol dehydrogenase 2; bile acid binding protein; 3-alpha hydroxysteroid dehydrogenase, type III) | 10 | 10p15-p14 | Metabolism of xenobiotics by cytochrome P450 | 0.11 | 0.3 |
| Hs.567256 |
| 10103 | 209114\_at | TSPAN1 | Hs.38972 | tetraspanin 1 | 1 | 1p34.1 | NA | 0.12 | 0.37 |
| 65125 | 211994\_at | WNK1 | Hs.356604 | WNK lysine deficient protein kinase 1 | 12 | 12p13.3 | NA | 0.12 | 0.23 |
| 587 | 203576\_at | BCAT2 | Hs.512670 | branched chain aminotransferase 2, mitochondrial | 19 | 19q13 | Valine, leucine and isoleucine degradation | 0.12 | 0.1 |
| Valine, leucine and isoleucine biosynthesis |
| Pantothenate and CoA biosynthesis |
| 6662 | 202936\_s\_at | SOX9 | Hs.642674 | SRY (sex determining region Y)-box 9 (campomelic dysplasia, autosomal sex-reversal) | 17 | 17q24.3-q25.1 | NA | 0.12 | 0.33 |
| 3603 | 209827\_s\_at | IL16 | Hs.459095 | interleukin 16 (lymphocyte chemoattractant factor) | 15 | 15q26.3 | NA | 0.12 | 0.37 |
| 1646 | 211653\_x\_at | AKR1C2 | Hs.460260 | aldo-keto reductase family 1, member C2 (dihydrodiol dehydrogenase 2; bile acid binding protein; 3-alpha hydroxysteroid dehydrogenase, type III) | 10 | 10p15-p14 | Metabolism of xenobiotics by cytochrome P450 | 0.12 | 0.33 |
| Hs.567256 |
| 59342 | 218217\_at | SCPEP1 | Hs.514950 | serine carboxypeptidase 1 | 17 | 17q22 | NA | 0.12 | 0.13 |
| 7295 | 216609\_at | TXN | Hs.435136 | thioredoxin | 9 | 9q31 | NA | 0.12 | 0.54 |
| 55119 | 218040\_at | PRPF38B | Hs.342307 | PRP38 pre-mRNA processing factor 38 (yeast) domain containing B | 1 | 1p13.3 | NA | 0.12 | 0.17 |
| 2805 | 208813\_at | GOT1 | Hs.500756 | glutamic-oxaloacetic transaminase 1, soluble (aspartate aminotransferase 1) | 10 | 10q24.1-q25.1 | Glutamate metabolism | 0.12 | 0 |
| Alanine and aspartate metabolism |
| Cysteine metabolism |
| Arginine and proline metabolism |
| Tyrosine metabolism |
| Phenylalanine metabolism |
| Phenylalanine, tyrosine and tryptophan biosynthesis |
| Novobiocin biosynthesis |
| Carbon fixation |
| Alkaloid biosynthesis I |
| 5476 | 200661\_at | PPGB | Hs.517076 | protective protein for beta-galactosidase (galactosialidosis) | 20 | 20q13.1 | NA | 0.12 | 0.27 |
| 25992 | 213493\_at | SNED1 | Hs.471834 | sushi, nidogen and EGF-like domains 1 | 2 | 2q37.3 | NA | 0.12 | 0.1 |
| 90925 | 210450\_at | LOC90925 | Hs.81221 | NA | 14 | 14q32.33 | NA | 0.12 | 0.06 |
| 6846 | 214567\_s\_at | XCL2 | Hs.458346 | chemokine (C motif) ligand 2 | 1 | 1q23-q25 | Cytokine-cytokine receptor interaction | 0.12 | 0.31 |
| Hs.546295 |
| 3004 | 207460\_at | GZMM | Hs.465511 | granzyme M (lymphocyte met-ase 1) | 19 | 19p13.3 | NA | 0.12 | 0.23 |
| 2206 | 207496\_at | MS4A2 | Hs.386748 | membrane-spanning 4-domains, subfamily A, member 2 (Fc fragment of IgE, high affinity I, receptor for; beta polypeptide) | 11 | 11q13 | Fc epsilon RI signaling pathway | 0.13 | 0.06 |
| 1329 | 213735\_s\_at | COX5B | Hs.1342 | cytochrome c oxidase subunit Vb | 2 | 2cen-q13 | Oxidative phosphorylation | 0.13 | 0.27 |
| 1804 | 215914\_at | DPP6 | Hs.334947 | dipeptidyl-peptidase 6 | 7 | 7q36.2 | NA | 0.13 | 0.4 |
| Hs.490684 |
| Hs.638892 |
| 28778 | 215035\_at | IGLV6-57 | Hs.535668 | immunoglobulin lambda variable 6-57 | 22 | 22q11.2 | NA | 0.13 | 0.27 |
| 11178 | 47550\_at | LZTS1 | Hs.521432 | leucine zipper, putative tumor suppressor 1 | 8 | 8p22 | NA | 0.13 | 0.37 |
| 3682 | 205055\_at | ITGAE | Hs.513867 | integrin, alpha E (antigen CD103, human mucosal lymphocyte antigen 1; alpha polypeptide) | 17 | 17p13 | Regulation of actin cytoskeleton | 0.13 | 0.47 |
| 5733 | 210834\_s\_at | PTGER3 | Hs.445000 | prostaglandin E receptor 3 (subtype EP3) | 1 | 1p31.2 | Calcium signaling pathway | 0.13 | 0 |
| Neuroactive ligand-receptor interaction |
| 3084 | 206343\_s\_at | NRG1 | Hs.453951 | neuregulin 1 | 8 | 8p21-p12 | NA | 0.13 | 0.17 |
| 1540 | 214272\_at | CYLD | Hs.578973 | cylindromatosis (turban tumor syndrome) | 16 | 16q12.1 | NA | 0.13 | 0.1 |
| 931 | 210356\_x\_at | MS4A1 | Hs.438040 | membrane-spanning 4-domains, subfamily A, member 1 | 11 | 11q12 | Hematopoietic cell lineage | 0.13 | 0.1 |
| 4673 | 208752\_x\_at | NAP1L1 | Hs.524599 | nucleosome assembly protein 1-like 1 | 12 | 12q21.2 | NA | 0.13 | 0.23 |
| Hs.643135 |
| 3495 | 214973\_x\_at | IGHD | Hs.510635 | immunoglobulin heavy constant delta | 14 | 14q32.33 | NA | 0.13 | 0.37 |
| 3695 | 205718\_at | ITGB7 | Hs.524458 | integrin, beta 7 | 12 | 12q13.13 | Focal adhesion | 0.13 | 0.23 |
| ECM-receptor interaction |
| Cell adhesion molecules (CAMs) |
| Regulation of actin cytoskeleton |
| 339562 | 217480\_x\_at | LOC339562 | Hs.449972 | NA | 2 | 2p11.1 | NA | 0.13 | 0.1 |
| 7993 | 215983\_s\_at | UBXD6 | Hs.153678 | UBX domain containing 6 | 8 | 8p12-p11.2 | NA | 0.13 | 0.13 |
| 2746 | 200947\_s\_at | GLUD1 | Hs.500409 | glutamate dehydrogenase 1 | 10 | 10q23.3 | Urea cycle and metabolism of amino groups | 0.13 | 0.33 |
| Glutamate metabolism |
| Arginine and proline metabolism |
| D-Glutamine and D-glutamate metabolism |
| Nitrogen metabolism |
| 1028 | 219534\_x\_at | CDKN1C | Hs.106070 | cyclin-dependent kinase inhibitor 1C (p57, Kip2) | 11 | 11p15.5 | Cell cycle | 0.13 | 0 |
| 55160 | 221656\_s\_at | ARHGEF10L | Hs.443460 | Rho guanine nucleotide exchange factor (GEF) 10-like | 1 | 1p36.13 | NA | 0.13 | 0.33 |
| 771 | 215867\_x\_at | CA12 | Hs.210995 | carbonic anhydrase XII | 15 | 15q22 | Nitrogen metabolism | 0.14 | 0.33 |
| 4285 | 204305\_at | MIPEP | Hs.507498 | mitochondrial intermediate peptidase | 13 | 13q12 | NA | 0.14 | 0.44 |
| 54805 | 206818\_s\_at | CNNM2 | Hs.643509 | cyclin M2 | 10 | 10q24.32 | NA | 0.14 | 0 |
| 912 | 205789\_at | CD1D | Hs.1799 | CD1d molecule | 1 | 1q22-q23 | Hematopoietic cell lineage | 0.14 | 0.31 |
| Hs.387539 |
| 5172 | 206529\_x\_at | SLC26A4 | Hs.571246 | solute carrier family 26, member 4 | 7 | 7q31 | NA | 0.14 | 0.1 |
| 916 | 205456\_at | CD3E | Hs.3003 | CD3e molecule, epsilon (CD3-TCR complex) | 11 | 11q23 | Hematopoietic cell lineage | 0.14 | 0.31 |
| T cell receptor signaling pathway |
| 2254 | 206404\_at | FGF9 | Hs.111 | fibroblast growth factor 9 (glia-activating factor) | 13 | 13q11-q12 | MAPK signaling pathway | 0.14 | 0.23 |
| Regulation of actin cytoskeleton |
| 266655 | 203245\_s\_at | FLJ35348 | Hs.592770 | NA | 9 | 9q34 | NA | 0.14 | 0.23 |
| 23216 | 212350\_at | TBC1D1 | Hs.176503 | TBC1 (tre-2/USP6, BUB2, cdc16) domain family, member 1 | 4 | 4p14 | NA | 0.14 | 0.1 |
| 191 | 200903\_s\_at | AHCY | Hs.388004 | S-adenosylhomocysteine hydrolase | 20 | 20cen-q13.1 | Methionine metabolism | 0.14 | 0.37 |
| Selenoamino acid metabolism |
| 9334 | 221485\_at | B4GALT5 | Hs.370487 | UDP-Gal:betaGlcNAc beta 1,4- galactosyltransferase, polypeptide 5 | 20 | 20q13.1-q13.2 | N-Glycan biosynthesis | 0.14 | 0.33 |
| Keratan sulfate biosynthesis |
| Glycan structures - biosynthesis 1 |
| 51606 | 221504\_s\_at | ATP6V1H | Hs.491737 | ATPase, H+ transporting, lysosomal 50/57kDa, V1 subunit H | 8 | 8p22-q22.3 | Oxidative phosphorylation | 0.14 | 0.31 |
| ATP synthesis |
| Cholera - Infection |
| Epithelial cell signaling in Helicobacter pylori infection |
| 9143 | 205691\_at | SYNGR3 | Hs.435277 | synaptogyrin 3 | 16 | 16p13 | NA | 0.14 | 0.33 |
| 5432 | 208996\_s\_at | POLR2C | Hs.79402 | polymerase (RNA) II (DNA directed) polypeptide C, 33kDa | 16 | 16q13-q21 | Purine metabolism | 0.14 | 0.5 |
| Pyrimidine metabolism |
| RNA polymerase |
| 6470 | 209980\_s\_at | SHMT1 | Hs.513987 | serine hydroxymethyltransferase 1 (soluble) | 17 | 17p11.2 | Glycine, serine and threonine metabolism | 0.14 | 0.31 |
| Hs.636044 | Lysine degradation |
| Hs.642675 | Cyanoamino acid metabolism |
|  | One carbon pool by folate |
|  | Methane metabolism |
| 54997 | 218872\_at | TESC | Hs.525709 | tescalcin | 12 | 12q24.22 | NA | 0.14 | 0.2 |
| 2745 | 206662\_at | GLRX | Hs.28988 | glutaredoxin (thioltransferase) | 5 | 5q14 | NA | 0.14 | 0.27 |
| 27324 | 215108\_x\_at | TNRC9 | Hs.460789 | trinucleotide repeat containing 9 | 16 | 16q12.1 | NA | 0.14 | 0.13 |
| 9842 | 212700\_x\_at | PLEKHM1 | Hs.514242 | pleckstrin homology domain containing, family M (with RUN domain) member 1 | 17 | 17q21.31 | NA | 0.14 | 0.37 |
| 80736 | 205597\_at | SLC44A4 | Hs.335355 | solute carrier family 44, member 4 | 6 | 6p21.3 | NA | 0.14 | 0.27 |
| 965 | 211744\_s\_at | CD58 | Hs.34341 | CD58 molecule | 1 | 1p13 | Cell adhesion molecules (CAMs) | 0.14 | 0.27 |
| 894 | 200953\_s\_at | CCND2 | Hs.376071 | cyclin D2 | 12 | 12p13 | Cell cycle | 0.14 | 0.1 |
| Wnt signaling pathway |
| Focal adhesion |
| Jak-STAT signaling pathway |
| Colorectal cancer |
| 1601 | 201279\_s\_at | DAB2 | Hs.481980 | disabled homolog 2, mitogen-responsive phosphoprotein (Drosophila) | 5 | 5p13 | NA | 0.14 | 0.1 |
| 6925 | 215164\_at | TCF4 | Hs.569908 | transcription factor 4 | 18 | 18q21.1 | NA | 0.14 | 0.06 |
| 23516 | 212110\_at | SLC39A14 | Hs.491232 | solute carrier family 39 (zinc transporter), member 14 | 8 | 8p21.3 | NA | 0.14 | 0.47 |
| 27075 | 217979\_at | TSPAN13 | Hs.364544 | tetraspanin 13 | 7 | 7p21.1 | NA | 0.14 | 0.31 |
| 11214 | 222024\_s\_at | AKAP13 | Hs.459211 | A kinase (PRKA) anchor protein 13 | 15 | 15q24-q25 | NA | 0.14 | 0.33 |
| 10099 | 200972\_at | TSPAN3 | Hs.5062 | tetraspanin 3 | 15 | 15q24.3 | NA | 0.14 | 0.27 |
| 9971 | 206340\_at | NR1H4 | Hs.282735 | nuclear receptor subfamily 1, group H, member 4 | 12 | 12q23.1 | NA | 0.14 | 0.44 |
| 79135 | 221620\_s\_at | FAM121B | Hs.495851 | family with sequence similarity 121B | X | Xp22.11 | NA | 0.14 | 0.33 |
| 23463 | 201609\_x\_at | ICMT | Hs.515688 | isoprenylcysteine carboxyl methyltransferase | 1 | 1p36.21 | NA | 0.14 | 0.17 |
| Hs.562083 |
| 51660 | 218024\_at | BRP44L | Hs.172755 | brain protein 44-like | 6 | 6q27 | NA | 0.14 | 0.5 |
| 1365 | 203953\_s\_at | CLDN3 | Hs.520943 | claudin 3 | 7 | 7q11.23 | Cell adhesion molecules (CAMs) | 0.14 | 0.2 |
| Tight junction |
| Leukocyte transendothelial migration |
| 79034 | 222002\_at | C7orf26 | Hs.487511 | chromosome 7 open reading frame 26 | 7 | 7p22.1 | NA | 0.14 | 0.17 |
| 622 | 211715\_s\_at | BDH1 | Hs.274539 | 3-hydroxybutyrate dehydrogenase, type 1 | 3 | 3q29 | Synthesis and degradation of ketone bodies | 0.14 | 0.37 |
| Butanoate metabolism |
| 783 | 213714\_at | CACNB2 | Hs.59093 | calcium channel, voltage-dependent, beta 2 subunit | 10 | 10p12 | MAPK signaling pathway | 0.14 | 0.32 |
| 54664 | 218930\_s\_at | TMEM106B | Hs.396358 | transmembrane protein 106B | 7 | 7p21.3 | NA | 0.14 | 0.54 |
|  | 205728\_at | NA | NA | NA | NA | NA | NA | 0.14 | 0.44 |
| 3624 | 210511\_s\_at | INHBA | Hs.583348 | inhibin, beta A (activin A, activin AB alpha polypeptide) | 7 | 7p15-p13 | Cytokine-cytokine receptor interaction | 0.14 | 0.1 |
| TGF-beta signaling pathway |
| 55753 | 219277\_s\_at | OGDHL | Hs.17860 | oxoglutarate dehydrogenase-like | 10 | 10q11.23 | Citrate cycle (TCA cycle) | 0.15 | 0.37 |
| Lysine degradation |
| Tryptophan metabolism |
| 7110 | 215855\_s\_at | TMF1 | Hs.267632 | TATA element modulatory factor 1 | 3 | 3p21-p12 | NA | 0.15 | 0.27 |
| Hs.595560 |
| 6819 | 205342\_s\_at | SULT1C1 | Hs.436123 | sulfotransferase family, cytosolic, 1C, member 1 | 2 | 2q11.1-q11.2 | Cysteine metabolism | 0.15 | 0.5 |
| 3500 | 217022\_s\_at | IGHG1 | Hs.510635 | immunoglobulin heavy constant gamma 1 (G1m marker) | 14 | 14q32.33 | NA | 0.15 | 0.1 |
| 2040 | 201061\_s\_at | STOM | Hs.253903 | stomatin | 9 | 9q34.1 | NA | 0.15 | 0.27 |
| 56548 | 206756\_at | CHST7 | Hs.129955 | carbohydrate (N-acetylglucosamine 6-O) sulfotransferase 7 | X | Xp11.23 | Chondroitin sulfate biosynthesis | 0.15 | 0.27 |
| Glycan structures - biosynthesis 1 |
| 2263 | 211401\_s\_at | FGFR2 | Hs.533683 | fibroblast growth factor receptor 2 (bacteria-expressed kinase, keratinocyte growth factor receptor, craniofacial dysostosis 1, Crouzon syndrome, Pfeiffer syndrome, Jackson-Weiss syndrome) | 10 | 10q26 | MAPK signaling pathway | 0.15 | 0.27 |
| Regulation of actin cytoskeleton |
| 23231 | 212311\_at | KIAA0746 | Hs.479384 | NA | 4 | 4p15.2 | NA | 0.15 | 0 |
| 1803 | 203717\_at | DPP4 | Hs.368912 | dipeptidyl-peptidase 4 (CD26, adenosine deaminase complexing protein 2) | 2 | 2q24.3 | NA | 0.15 | 0.27 |
| 64499 | 207741\_x\_at | TPSB2 | Hs.405479 | tryptase beta 2 | 16 | 16p13.3 | NA | 0.15 | 0.06 |
| Hs.592982 |
| 23560 | 218238\_at | GTPBP4 | Hs.215766 | GTP binding protein 4 | 10 | 10p15-p14 | NA | 0.15 | 0 |
| 23225 | 220035\_at | NUP210 | Hs.475525 | nucleoporin 210kDa | 3 | 3p25.1 | NA | 0.15 | 0.1 |
| 26279 | 220423\_at | PLA2G2D | Hs.189507 | phospholipase A2, group IID | 1 | 1p36.12 | Glycerophospholipid metabolism | 0.15 | 0.27 |
| Arachidonic acid metabolism |
| Linoleic acid metabolism |
| MAPK signaling pathway |
| VEGF signaling pathway |
| Fc epsilon RI signaling pathway |
| Long-term depression |
| GnRH signaling pathway |
| 55793 | 221856\_s\_at | FAM63A | Hs.3346 | family with sequence similarity 63, member A | 1 | 1q21.2 | NA | 0.15 | 0 |
| 54910 | 46665\_at | SEMA4C | Hs.516220 | sema domain, immunoglobulin domain (Ig), transmembrane domain (TM) and short cytoplasmic domain, (semaphorin) 4C | 2 | 2q11.2 | Axon guidance | 0.15 | 0.06 |
| 183 | 202834\_at | AGT | Hs.19383 | angiotensinogen (serpin peptidase inhibitor, clade A, member 8) | 1 | 1q42-q43 | NA | 0.15 | 0.1 |
| 3856 | 209008\_x\_at | KRT8 | Hs.533782 | keratin 8 | 12 | 12q13 | Cell Communication | 0.15 | 0.17 |
| Hs.596312 |
| 6401 | 206211\_at | SELE | Hs.89546 | selectin E (endothelial adhesion molecule 1) | 1 | 1q22-q25 | Cell adhesion molecules (CAMs) | 0.15 | 0.1 |
| 2115 | 221911\_at | ETV1 | Hs.22634 | ets variant gene 1 | 7 | 7p21.3 | NA | 0.15 | 0.17 |
| 143 | 202239\_at | PARP4 | Hs.591227 | poly (ADP-ribose) polymerase family, member 4 | 13 | 13q11 | NA | 0.15 | 0.26 |
| 5303 | 214224\_s\_at | PIN4 | Hs.118076 | protein (peptidylprolyl cis/trans isomerase) NIMA-interacting, 4 (parvulin) | X | Xq13 | NA | 0.15 | 0.23 |
| 2017 | 214073\_at | CTTN | Hs.632133 | cortactin | 11 | 11q13 | Tight junction | 0.15 | 0.17 |
| Pathogenic Escherichia coli infection - EHEC |
| Pathogenic Escherichia coli infection - EPEC |
| 80760 | 219064\_at | ITIH5 | Hs.498586 | inter-alpha (globulin) inhibitor H5 | 10 | 10p14 | NA | 0.15 | 0.23 |
| 54463 | 213663\_s\_at | FLJ20152 | Hs.481704 | NA | 5 | 5p15.1 | NA | 0.15 | 0.27 |
|  | 202969\_at | NA | NA | NA | NA | NA | NA | 0.15 | 0.23 |
| 140462 | 205673\_s\_at | ASB9 | Hs.19404 | ankyrin repeat and SOCS box-containing 9 | X | NA | NA | 0.15 | 0.5 |
| 3125 | 221491\_x\_at | HLA-DRB3 | Hs.534322 | major histocompatibility complex, class II, DR beta 3 | 6 | 6p21.3 | Cell adhesion molecules (CAMs) | 0.15 | 0.27 |
| Antigen processing and presentation |
| Hematopoietic cell lineage |
| Type I diabetes mellitus |
| 3329 | 200807\_s\_at | HSPD1 | Hs.595053 | heat shock 60kDa protein 1 (chaperonin) | 2 | 2q33.1 | Type I diabetes mellitus | 0.15 | 0.17 |
| Hs.632539 | Prion disease |
| 6094 | 205806\_at | ROM1 | Hs.281564 | retinal outer segment membrane protein 1 | 11 | 11q13 | NA | 0.15 | 0.1 |
| 3707 | 203723\_at | ITPKB | Hs.528087 | inositol 1,4,5-trisphosphate 3-kinase B | 1 | 1q42.13 | Inositol phosphate metabolism | 0.15 | 0.13 |
| Calcium signaling pathway |
| Phosphatidylinositol signaling system |
|  | 212993\_at | NA | NA | NA | NA | NA | NA | 0.15 | 0.33 |
| 1152 | 200884\_at | CKB | Hs.173724 | creatine kinase, brain | 14 | 14q32 | Urea cycle and metabolism of amino groups | 0.15 | 0.2 |
| Arginine and proline metabolism |
| 1350 | 201134\_x\_at | COX7C | Hs.430075 | cytochrome c oxidase subunit VIIc | 5 | 5q14 | Oxidative phosphorylation | 0.15 | 0.23 |
| 6775 | 206118\_at | STAT4 | Hs.80642 | signal transducer and activator of transcription 4 | 2 | 2q32.2-q32.3 | Jak-STAT signaling pathway | 0.15 | 0.06 |
| 7380 | 206771\_at | UPK3A | Hs.632787 | uroplakin 3A | 22 | 22q13.31 | NA | 0.15 | 0.17 |
| 55603 | 221766\_s\_at | FAM46A | Hs.10784 | family with sequence similarity 46, member A | 6 | 6q14 | NA | 0.15 | 0.41 |
| 10991 | 205972\_at | SLC38A3 | Hs.76460 | solute carrier family 38, member 3 | 3 | 3p21.3 | NA | 0.15 | 0.23 |
| 9223 | 222372\_at | MAGI1 | Hs.476636 | membrane associated guanylate kinase, WW and PDZ domain containing 1 | 3 | 3p14.1 | Tight junction | 0.15 | 0.23 |
| Dentatorubropallidoluysian atrophy (DRPLA) |
| 7873 | 202655\_at | ARMET | Hs.436446 | arginine-rich, mutated in early stage tumors | 3 | 3p21.1 | NA | 0.15 | 0.23 |
| 9595 | 209606\_at | PSCDBP | Hs.270 | pleckstrin homology, Sec7 and coiled-coil domains, binding protein | 2 | 2q11.2 | NA | 0.15 | 0.1 |
| 2908 | 201866\_s\_at | NR3C1 | Hs.122926 | nuclear receptor subfamily 3, group C, member 1 (glucocorticoid receptor) | 5 | 5q31.3 | Neuroactive ligand-receptor interaction | 0.15 | 0.2 |
| 10613 | 202441\_at | SPFH1 | Hs.150087 | SPFH domain family, member 1 | 10 | 10q21-q22 | NA | 0.15 | 0.17 |
| 51117 | 218328\_at | COQ4 | Hs.98541 | coenzyme Q4 homolog (S. cerevisiae) | 9 | 9q34.11 | NA | 0.15 | 0.13 |
| 11027 | 211102\_s\_at | LILRA2 | Hs.534394 | leukocyte immunoglobulin-like receptor, subfamily A (with TM domain), member 2 | 19 | 19q13.4 | NA | 0.15 | 0.19 |
| 149478 | 215462\_at | LOC149478 | Hs.632400 | NA | 1 | 1p34.1 | NA | 0.15 | 0.17 |
| 2975 | 35671\_at | GTF3C1 | Hs.371718 | general transcription factor IIIC, polypeptide 1, alpha 220kDa | 16 | 16p12 | NA | 0.15 | 0.33 |
| 10768 | 200850\_s\_at | AHCYL1 | Hs.485365 | S-adenosylhomocysteine hydrolase-like 1 | 1 | 1p13.2 | Methionine metabolism | 0.15 | 0.13 |
| Hs.592725 | Selenoamino acid metabolism |
| 2239 | 204983\_s\_at | GPC4 | Hs.58367 | glypican 4 | X | Xq26.1 | NA | 0.15 | 0.2 |
| 9478 | 208320\_at | CABP1 | Hs.458482 | calcium binding protein 1 (calbrain) | 12 | 12q24.31 | NA | 0.16 | 0.19 |
| 79840 | 219418\_at | NHEJ1 | Hs.225988 | nonhomologous end-joining factor 1 | 2 | 2q35 | NA | 0.16 | 0.1 |
| 5627 | 207808\_s\_at | PROS1 | Hs.64016 | protein S (alpha) | 3 | 3q11.2 | Complement and coagulation cascades | 0.16 | 0.06 |
| 79154 | 218756\_s\_at | MGC4172 | Hs.462859 | NA | 17 | 17q12 | NA | 0.16 | 0.17 |
| 10787 | 207738\_s\_at | NCKAP1 | Hs.516633 | NCK-associated protein 1 | 2 | 2q32 | Regulation of actin cytoskeleton | 0.16 | 0.17 |
| 3418 | 210045\_at | IDH2 | Hs.513141 | isocitrate dehydrogenase 2 (NADP+), mitochondrial | 15 | 15q26.1 | Citrate cycle (TCA cycle) | 0.16 | 0.23 |
| Glutathione metabolism |
| Reductive carboxylate cycle (CO2 fixation) |
| 9910 | 213982\_s\_at | RABGAP1L | Hs.585378 | RAB GTPase activating protein 1-like | 1 | 1q24 | NA | 0.16 | 0.1 |
| Hs.591475 |
| Hs.615081 |
| 57473 | 55872\_at | GM632 | Hs.551552 | NA | 20 | 20q13.33 | NA | 0.16 | 0.13 |
| 23244 | 213984\_at | SCC-112 | Hs.331431 | NA | 4 | 4p14 | NA | 0.16 | 0.27 |
| 347733 | 214023\_x\_at | TUBB2B | Hs.300701 | tubulin, beta 2B | 6 | 6p25 | Gap junction | 0.16 | 0.23 |
| Pathogenic Escherichia coli infection - EHEC |
| Pathogenic Escherichia coli infection - EPEC |
| 7525 | 202933\_s\_at | YES1 | Hs.194148 | v-yes-1 Yamaguchi sarcoma viral oncogene homolog 1 | 18 | 18p11.31-p11.21 | Adherens junction | 0.16 | 0.5 |
| Tight junction |
| 7994 | 202423\_at | MYST3 | Hs.591848 | MYST histone acetyltransferase (monocytic leukemia) 3 | 8 | 8p11 | Valine, leucine and isoleucine degradation | 0.16 | 0 |
| Tyrosine metabolism |
| Phenylalanine metabolism |
| Glycerophospholipid metabolism |
| 1- and 2-Methylnaphthalene degradation |
| Benzoate degradation via CoA ligation |
| Ethylbenzene degradation |
| Limonene and pinene degradation |
| Alkaloid biosynthesis II |
| 10102 | 212656\_at | TSFM | Hs.632704 | Ts translation elongation factor, mitochondrial | 12 | 12q13-q14 | NA | 0.16 | 0.23 |
| 93973 | 218658\_s\_at | ACTR8 | Hs.412186 | ARP8 actin-related protein 8 homolog (yeast) | 3 | NA | NA | 0.16 | 0.06 |
| 53335 | 219497\_s\_at | BCL11A | Hs.370549 | B-cell CLL/lymphoma 11A (zinc finger protein) | 2 | 2p16.1 | NA | 0.16 | 0.37 |
| 1203 | 204084\_s\_at | CLN5 | Hs.30213 | ceroid-lipofuscinosis, neuronal 5 | 13 | 13q21.1-q32 | NA | 0.16 | 0.13 |
| 9601 | 211048\_s\_at | PDIA4 | Hs.93659 | protein disulfide isomerase family A, member 4 | 7 | 7q35 | Cholera - Infection | 0.16 | 0.33 |
| 28959 | 220532\_s\_at | LR8 | Hs.438823 | NA | 7 | 7q36.1 | NA | 0.16 | 0.06 |
| 114884 | 219073\_s\_at | OSBPL10 | Hs.150122 | oxysterol binding protein-like 10 | 3 | 3p22.3 | NA | 0.16 | 0.5 |
| 3426 | 203854\_at | CFI | Hs.312485 | complement factor I | 4 | 4q25 | Complement and coagulation cascades | 0.16 | 0.4 |
| 2571 | 205278\_at | GAD1 | Hs.420036 | glutamate decarboxylase 1 (brain, 67kDa) | 2 | 2q31 | Glutamate metabolism | 0.16 | 0.13 |
| Alanine and aspartate metabolism |
| beta-Alanine metabolism |
| Taurine and hypotaurine metabolism |
| Butanoate metabolism |
| Type I diabetes mellitus |
| 79004 | 218097\_s\_at | CUEDC2 | Hs.500874 | CUE domain containing 2 | 10 | 10q24.32 | NA | 0.16 | 0.23 |
| 1351 | 201119\_s\_at | COX8A | Hs.433901 | cytochrome c oxidase subunit 8A (ubiquitous) | 11 | 11q12-q13 | Oxidative phosphorylation | 0.16 | 0.17 |
| 2585 | 205219\_s\_at | GALK2 | Hs.122006 | galactokinase 2 | 15 | 15q21.1 | Galactose metabolism | 0.16 | 0.5 |
| Hs.643456 |
| 1495 | 210844\_x\_at | CTNNA1 | Hs.534797 | catenin (cadherin-associated protein), alpha 1, 102kDa | 5 | 5q31 | Adherens junction | 0.16 | 0.2 |
| Tight junction |
| Leukocyte transendothelial migration |
| 1244 | 206155\_at | ABCC2 | Hs.368243 | ATP-binding cassette, sub-family C (CFTR/MRP), member 2 | 10 | 10q24 | ABC transporters - General | 0.16 | 0.2 |
| 84263 | 215436\_at | HSDL2 | Hs.59486 | hydroxysteroid dehydrogenase like 2 | 9 | 9q32 | NA | 0.16 | 0.13 |
| 27244 | 218346\_s\_at | SESN1 | Hs.591336 | sestrin 1 | 6 | 6q21 | NA | 0.16 | 0.37 |
| 2271 | 214170\_x\_at | FH | Hs.498239 | fumarate hydratase | 1 | 1q42.1 | Citrate cycle (TCA cycle) | 0.16 | 0.33 |
| Reductive carboxylate cycle (CO2 fixation) |
| 4891 | 203125\_x\_at | SLC11A2 | Hs.505545 | solute carrier family 11 (proton-coupled divalent metal ion transporters), member 2 | 12 | 12q13 | NA | 0.16 | 0.13 |
| 7692 | 216960\_s\_at | ZNF133 | Hs.472221 | zinc finger protein 133 | 20 | 20p11.23-20p11.22 | NA | 0.16 | 0.81 |
| 9788 | 203037\_s\_at | MTSS1 | Hs.336994 | metastasis suppressor 1 | 8 | 8p22 | NA | 0.16 | 0.4 |
| 2186 | 209271\_at | FALZ | Hs.444200 | fetal Alzheimer antigen | 17 | 17q24.3 | NA | 0.16 | 0.06 |
| 1454 | 202332\_at | CSNK1E | Hs.474833 | casein kinase 1, epsilon | 22 | 22q13.1 | Wnt signaling pathway | 0.16 | 0.06 |
| Hedgehog signaling pathway |
| Gap junction |
| Circadian rhythm |
| 10591 | 204238\_s\_at | C6orf108 | Hs.109752 | chromosome 6 open reading frame 108 | 6 | 6p21.1 | NA | 0.16 | 0.17 |
| 1645 | 216594\_x\_at | AKR1C1 | Hs.460260 | aldo-keto reductase family 1, member C1 (dihydrodiol dehydrogenase 1; 20-alpha (3-alpha)-hydroxysteroid dehydrogenase) | 10 | 10p15-p14 | Metabolism of xenobiotics by cytochrome P450 | 0.16 | 0.17 |
| Hs.567256 |
| 6590 | 203021\_at | SLPI | Hs.517070 | secretory leukocyte peptidase inhibitor | 20 | 20q12 | NA | 0.16 | 0.17 |
| 2542 | 202830\_s\_at | SLC37A4 | Hs.132760 | solute carrier family 37 (glycerol-6-phosphate transporter), member 4 | 11 | 11q23.3 | NA | 0.16 | 0.31 |
| 5796 | 203038\_at | PTPRK | Hs.155919 | protein tyrosine phosphatase, receptor type, K | 6 | 6q22.2-23.1 | NA | 0.16 | 0.5 |
| 81839 | 219330\_at | VANGL1 | Hs.515130 | vang-like 1 (van gogh, Drosophila) | 1 | 1p11-p13.1 | Wnt signaling pathway | 0.16 | 0.06 |
| 1622 | 211070\_x\_at | DBI | Hs.78888 | diazepam binding inhibitor (GABA receptor modulator, acyl-Coenzyme A binding protein) | 2 | 2q12-q21 | PPAR signaling pathway | 0.16 | 0.17 |
| 6391 | 216591\_s\_at | SDHC | Hs.444472 | succinate dehydrogenase complex, subunit C, integral membrane protein, 15kDa | 1 | 1q21 | Citrate cycle (TCA cycle) | 0.16 | 0.13 |
| Oxidative phosphorylation |
| 57787 | 55065\_at | MARK4 | Hs.34314 | MAP/microtubule affinity-regulating kinase 4 | 19 | 19q13.3 | NA | 0.16 | 0.7 |
| 7832 | 201236\_s\_at | BTG2 | Hs.519162 | BTG family, member 2 | 1 | 1q32 | NA | 0.16 | 0.23 |
| 8775 | 206491\_s\_at | NAPA | Hs.126938 | N-ethylmaleimide-sensitive factor attachment protein, alpha | 19 | 19q13.32 | NA | 0.16 | 0.06 |
| 11145 | 209581\_at | HRASLS3 | Hs.502775 | HRAS-like suppressor 3 | 11 | 11q12.3-q13.1 | NA | 0.16 | 0.31 |
| 79893 | 218079\_s\_at | ZNF403 | Hs.514116 | zinc finger protein 403 | 17 | 17q12 | NA | 0.16 | 0.33 |
| 55294 | 218751\_s\_at | FBXW7 | Hs.561245 | F-box and WD-40 domain protein 7 (archipelago homolog, Drosophila) | 4 | 4q31.3 | Neurodegenerative Disorders | 0.16 | 0.17 |
| Ubiquitin mediated proteolysis |
| 3398 | 213931\_at | ID2 | Hs.180919 | inhibitor of DNA binding 2, dominant negative helix-loop-helix protein | 2 | 2p25 | TGF-beta signaling pathway | 0.16 | 0.44 |
| 653249 | 215470\_at | DKFZP686P18101 | Hs.202179 | NA | 5 | 5q13.2 | NA | 0.16 | 0.3 |
| 26999 | 220999\_s\_at | CYFIP2 | Hs.519702 | cytoplasmic FMR1 interacting protein 2 | 5 | 5q33.3 | Regulation of actin cytoskeleton | 0.16 | 0.33 |
| 6229 | 200061\_s\_at | RPS24 | Hs.356794 | ribosomal protein S24 | 10 | 10q22-q23 | Ribosome | 0.16 | 0.1 |
| 894 | 200952\_s\_at | CCND2 | Hs.376071 | cyclin D2 | 12 | 12p13 | Cell cycle | 0.16 | 0.1 |
| Wnt signaling pathway |
| Focal adhesion |
| Jak-STAT signaling pathway |
| Colorectal cancer |
| 4050 | 207339\_s\_at | LTB | Hs.376208 | lymphotoxin beta (TNF superfamily, member 3) | 6 | 6p21.3 | Cytokine-cytokine receptor interaction | 0.16 | 0.27 |
|  | 217556\_at | NA | NA | NA | NA | NA | NA | 0.16 | 0.27 |
| 1004 | 205533\_s\_at | CDH6 | Hs.171054 | cadherin 6, type 2, K-cadherin (fetal kidney) | 5 | 5p15.1-p14 | NA | 0.16 | 0.17 |
| 1080 | 215702\_s\_at | CFTR | Hs.489786 | cystic fibrosis transmembrane conductance regulator, ATP-binding cassette (sub-family C, member 7) | 7 | 7q31.2 | ABC transporters - General | 0.16 | 0.31 |
| Hs.621460 |
| 1346 | 204570\_at | COX7A1 | Hs.421621 | cytochrome c oxidase subunit VIIa polypeptide 1 (muscle) | 19 | 19q13.1 | Oxidative phosphorylation | 0.16 | 0.23 |
| Hs.631480 |
| 56270 | 209076\_s\_at | WDR45L | Hs.132161 | WDR45-like | 17 | 17q25.3 | NA | 0.16 | 0.06 |
| 6003 | 210258\_at | RGS13 | Hs.497220 | regulator of G-protein signalling 13 | 1 | 1q31.2 | NA | 0.16 | 0.13 |
| 8558 | 210622\_x\_at | CDK10 | Hs.109 | cyclin-dependent kinase (CDC2-like) 10 | 16 | 16q24 | NA | 0.16 | 0.27 |
| 8473 | 209240\_at | OGT | Hs.405410 | O-linked N-acetylglucosamine (GlcNAc) transferase (UDP-N-acetylglucosamine:polypeptide-N-acetylglucosaminyl transferase) | X | Xq13 | Fructose and mannose metabolism | 0.16 | 0.1 |
| Glycerolipid metabolism |
| Glycan structures - biosynthesis 2 |
| 1236 | 206337\_at | CCR7 | Hs.370036 | chemokine (C-C motif) receptor 7 | 17 | 17q12-q21.2 | Cytokine-cytokine receptor interaction | 0.16 | 0.17 |
| 3418 | 210046\_s\_at | IDH2 | Hs.513141 | isocitrate dehydrogenase 2 (NADP+), mitochondrial | 15 | 15q26.1 | Citrate cycle (TCA cycle) | 0.16 | 0.17 |
| Glutathione metabolism |
| Reductive carboxylate cycle (CO2 fixation) |
| 515 | 211755\_s\_at | ATP5F1 | Hs.514870 | ATP synthase, H+ transporting, mitochondrial F0 complex, subunit B1 | 1 | 1p13.2 | Oxidative phosphorylation | 0.16 | 0.37 |
| ATP synthesis |
| 117178 | 203019\_x\_at | SSX2IP | Hs.22587 | synovial sarcoma, X breakpoint 2 interacting protein | 1 | 1p22.3 | Adherens junction | 0.16 | 0.33 |
| 23371 | 212494\_at | TENC1 | Hs.6147 | tensin like C1 domain containing phosphatase (tensin 2) | 12 | 12q13.13 | NA | 0.16 | 0.3 |
| 3633 | 213804\_at | INPP5B | Hs.449942 | inositol polyphosphate-5-phosphatase, 75kDa | 1 | 1p34 | Inositol phosphate metabolism | 0.16 | 0.06 |
| Phosphatidylinositol signaling system |
| 4057 | 202018\_s\_at | LTF | Hs.529517 | lactotransferrin | 3 | 3q21-q23 | NA | 0.16 | 0.44 |
| 57017 | 212228\_s\_at | COQ9 | Hs.513632 | coenzyme Q9 homolog (S. cerevisiae) | 16 | 16q13 | NA | 0.16 | 0 |
| 5797 | 203329\_at | PTPRM | Hs.49774 | protein tyrosine phosphatase, receptor type, M | 18 | 18p11.2 | Cell adhesion molecules (CAMs) | 0.16 | 0.17 |
| Adherens junction |
| 10010 | 207616\_s\_at | TANK | Hs.556496 | TRAF family member-associated NFKB activator | 2 | 2q24-q31 | NA | 0.16 | 0 |
| 133 | 202912\_at | ADM | Hs.441047 | adrenomedullin | 11 | 11p15.4 | NA | 0.16 | 0 |
| 22981 | 207705\_s\_at | RP4-691N24.1 | Hs.472347 | NA | 20 | 20p11.22-p11.1 | NA | 0.16 | 0.33 |
| 4293 | 213927\_at | MAP3K9 | Hs.445496 | mitogen-activated protein kinase kinase kinase 9 | 14 | 14q24.3-q31 | NA | 0.16 | 0.13 |
| Hs.593542 |
| 93487 | 212643\_at | C14orf32 | Hs.594338 | chromosome 14 open reading frame 32 | 14 | 14q22.3 | NA | 0.16 | 0.23 |
| 5583 | 218764\_at | PRKCH | Hs.333907 | protein kinase C, eta | 14 | 14q22-q23 | Tight junction | 0.16 | 0.06 |
| 80150 | 218857\_s\_at | ASRGL1 | Hs.535326 | asparaginase like 1 | 11 | 11q12.3 | NA | 0.16 | 0.44 |
| 629 | 211920\_at | CFB | Hs.69771 | complement factor B | 6 | 6p21.3 | Complement and coagulation cascades | 0.16 | 0.23 |
| 1160 | 205295\_at | CKMT2 | Hs.80691 | creatine kinase, mitochondrial 2 (sarcomeric) | 5 | 5q13.3 | Urea cycle and metabolism of amino groups | 0.17 | 0.31 |
| Arginine and proline metabolism |
| 23382 | 212814\_at | KIAA0828 | Hs.600789 | NA | 7 | 7q32.1 | Methionine metabolism | 0.17 | 0.44 |
| Selenoamino acid metabolism |
| 28461 | 211635\_x\_at | IGHV1-69 | Hs.634941 | immunoglobulin heavy variable 1-69 | 14 | 14q32.32-q32.33 | NA | 0.17 | 0.1 |
| 771 | 214164\_x\_at | CA12 | Hs.210995 | carbonic anhydrase XII | 15 | 15q22 | Nitrogen metabolism | 0.17 | 0.44 |
| 80704 | 220736\_at | SLC19A3 | Hs.221597 | solute carrier family 19, member 3 | 2 | 2q37 | NA | 0.17 | 0.1 |
| 1677 | 206752\_s\_at | DFFB | Hs.133089 | DNA fragmentation factor, 40kDa, beta polypeptide (caspase-activated DNase) | 1 | 1p36.3 | Apoptosis | 0.17 | 0.17 |
| 9700 | 204817\_at | ESPL1 | Hs.153479 | extra spindle poles like 1 (S. cerevisiae) | 12 | 12q | Cell cycle | 0.17 | 0.27 |
| 64866 | 218451\_at | CDCP1 | Hs.476093 | CUB domain containing protein 1 | 3 | 3p21.31 | NA | 0.17 | 0.3 |
| 6455 | 201851\_at | SH3GL1 | Hs.97616 | SH3-domain GRB2-like 1 | 19 | 19p13.3 | NA | 0.17 | 0.37 |
| 26097 | 202559\_x\_at | C1orf77 | Hs.371788 | chromosome 1 open reading frame 77 | 1 | 1q21.3 | NA | 0.17 | 0 |
| 64398 | 219321\_at | MPP5 | Hs.642697 | membrane protein, palmitoylated 5 (MAGUK p55 subfamily member 5) | 14 | 14q23.3 | Tight junction | 0.17 | 0.17 |
| 3434 | 203153\_at | IFIT1 | Hs.20315 | interferon-induced protein with tetratricopeptide repeats 1 | 10 | 10q25-q26 | NA | 0.17 | 0.13 |
| 23508 | 213174\_at | TTC9 | Hs.79170 | tetratricopeptide repeat domain 9 | 14 | 14q24.2 | NA | 0.17 | 0.06 |
| 54458 | 217794\_at | PRR13 | Hs.426359 | proline rich 13 | 12 | 12q12 | NA | 0.17 | 0 |
| Hs.631599 |
| 55759 | 218512\_at | WDR12 | Hs.73291 | WD repeat domain 12 | 2 | 2q33.1 | NA | 0.17 | 0.23 |
| 10061 | 207622\_s\_at | ABCF2 | Hs.438823 | ATP-binding cassette, sub-family F (GCN20), member 2 | 7 | 7q36 | NA | 0.17 | 0.27 |
| 535 | 212383\_at | ATP6V0A1 | Hs.463074 | ATPase, H+ transporting, lysosomal V0 subunit a1 | 17 | 17q21 | Oxidative phosphorylation | 0.17 | 0.17 |
| ATP synthesis |
| Cholera - Infection |
| Epithelial cell signaling in Helicobacter pylori infection |
| 3676 | 213416\_at | ITGA4 | Hs.440955 | integrin, alpha 4 (antigen CD49D, alpha 4 subunit of VLA-4 receptor) | 2 | 2q31.3 | Focal adhesion | 0.17 | 0 |
| ECM-receptor interaction |
| Cell adhesion molecules (CAMs) |
| Hematopoietic cell lineage |
| Leukocyte transendothelial migration |
| Regulation of actin cytoskeleton |
| 26525 | 222223\_s\_at | IL1F5 | Hs.516301 | interleukin 1 family, member 5 (delta) | 2 | 2q14 | NA | 0.17 | 0.1 |
| 2843 | 214510\_at | GPR20 | Hs.188859 | G protein-coupled receptor 20 | 8 | 8q24.2-q24.3 | NA | 0.17 | 0 |
| 54101 | 221215\_s\_at | RIPK4 | Hs.517310 | receptor-interacting serine-threonine kinase 4 | 21 | 21q22.3 | NA | 0.17 | 0.44 |
| 4318 | 203936\_s\_at | MMP9 | Hs.297413 | matrix metallopeptidase 9 (gelatinase B, 92kDa gelatinase, 92kDa type IV collagenase) | 20 | 20q11.2-q13.1 | Leukocyte transendothelial migration | 0.17 | 0 |
| 8202 | 209062\_x\_at | NCOA3 | Hs.592142 | nuclear receptor coactivator 3 | 20 | 20q12 | NA | 0.17 | 0.06 |
| 1359 | 205624\_at | CPA3 | Hs.646 | carboxypeptidase A3 (mast cell) | 3 | 3q21-q25 | NA | 0.17 | 0.17 |
|  | 216558\_x\_at | NA | NA | NA | NA | NA | NA | 0.17 | 0.2 |
| 23365 | 201335\_s\_at | ARHGEF12 | Hs.24598 | Rho guanine nucleotide exchange factor (GEF) 12 | 11 | 11q23.3 | Axon guidance | 0.17 | 0.27 |
| Regulation of actin cytoskeleton |
| 224 | 202054\_s\_at | ALDH3A2 | Hs.499886 | aldehyde dehydrogenase 3 family, member A2 | 17 | 17p11.2 | Glycolysis / Gluconeogenesis | 0.17 | 0.37 |
| Ascorbate and aldarate metabolism |
| Fatty acid metabolism |
| Bile acid biosynthesis |
| Valine, leucine and isoleucine degradation |
| Lysine degradation |
| Arginine and proline metabolism |
| Histidine metabolism |
| Tryptophan metabolism |
| beta-Alanine metabolism |
| Glycerolipid metabolism |
| Pyruvate metabolism |
| Propanoate metabolism |
| Butanoate metabolism |
| Limonene and pinene degradation |
| 25978 | 202538\_s\_at | CHMP2B | Hs.476930 | chromatin modifying protein 2B | 3 | 3p11.2 | NA | 0.17 | 0.17 |
| 9760 | 204529\_s\_at | TOX | Hs.491805 | NA | 8 | 8q12.1 | NA | 0.17 | 0.23 |
| Hs.634856 |
| 65009 | 209159\_s\_at | NDRG4 | Hs.322430 | NDRG family member 4 | 16 | 16q21-q22.1 | NA | 0.17 | 0.06 |
| 8718 | 211841\_s\_at | TNFRSF25 | Hs.462529 | tumor necrosis factor receptor superfamily, member 25 | 1 | 1p36.2 | Cytokine-cytokine receptor interaction | 0.17 | 0.13 |
| 827 | 217387\_at | CAPN6 | Hs.496593 | calpain 6 | X | Xq23 | NA | 0.17 | 0.06 |
| 10467 | 201541\_s\_at | ZNHIT1 | Hs.211079 | zinc finger, HIT type 1 | 7 | 7q22.1 | NA | 0.17 | 0.23 |
| 54951 | 218351\_at | COMMD8 | Hs.23956 | COMM domain containing 8 | 4 | 4p12 | NA | 0.17 | 0.33 |
| 35 | 202366\_at | ACADS | Hs.507076 | acyl-Coenzyme A dehydrogenase, C-2 to C-3 short chain | 12 | 12q22-qter | Fatty acid metabolism | 0.17 | 0.33 |
| Valine, leucine and isoleucine degradation |
| Butanoate metabolism |
| 10579 | 211382\_s\_at | TACC2 | Hs.501252 | transforming, acidic coiled-coil containing protein 2 | 10 | 10q26 | NA | 0.17 | 0.44 |
| Hs.643068 |
| 527 | 36994\_at | ATP6V0C | Hs.389107 | ATPase, H+ transporting, lysosomal 16kDa, V0 subunit c | 16 | 16p13.3 | Oxidative phosphorylation | 0.17 | 0.2 |
| ATP synthesis |
| Cholera - Infection |
| Epithelial cell signaling in Helicobacter pylori infection |
| 23774 | 204520\_x\_at | BRD1 | Hs.127950 | bromodomain containing 1 | 22 | 22q13.33 | NA | 0.17 | 0.33 |
| 3397 | 208937\_s\_at | ID1 | Hs.504609 | inhibitor of DNA binding 1, dominant negative helix-loop-helix protein | 20 | 20q11 | TGF-beta signaling pathway | 0.17 | 0.27 |
| 3094 | 207721\_x\_at | HINT1 | Hs.483305 | histidine triad nucleotide binding protein 1 | 5 | 5q31.2 | NA | 0.17 | 0.17 |
| 22902 | 213430\_at | RUFY3 | Hs.7972 | RUN and FYVE domain containing 3 | 4 | 4q13.3 | NA | 0.17 | 0.23 |
| 533 | 200078\_s\_at | ATP6V0B | Hs.632406 | ATPase, H+ transporting, lysosomal 21kDa, V0 subunit b | 1 | 1p32.3 | Oxidative phosphorylation | 0.17 | 0.37 |
| ATP synthesis |
| Cholera - Infection |
| Epithelial cell signaling in Helicobacter pylori infection |
|  | 217412\_at | NA | NA | NA | NA | NA | NA | 0.17 | 0.13 |
| 10578 | 37145\_at | GNLY | Hs.105806 | granulysin | 2 | 2p12-q11 | NA | 0.17 | 0.17 |
| 2620 | 205848\_at | GAS2 | Hs.523543 | growth arrest-specific 2 | 11 | 11p14.3-p15.2 | NA | 0.17 | 0.33 |
| 2866 | 221385\_s\_at | GPR42 | Hs.533926 | G protein-coupled receptor 42 | 19 | 19q13.1 | NA | 0.17 | 0.27 |
| 22928 | 200961\_at | SEPHS2 | Hs.118725 | selenophosphate synthetase 2 | 16 | 16p11.2 | Selenoamino acid metabolism | 0.17 | 0 |
| 23001 | 212598\_at | WDFY3 | Hs.480116 | WD repeat and FYVE domain containing 3 | 4 | 4q21.23 | NA | 0.17 | 0.33 |
| 23710 | 208869\_s\_at | GABARAPL1 | Hs.524250 | GABA(A) receptor-associated protein like 1 | 12 | 12p13.2 | Regulation of autophagy | 0.17 | 0.33 |
| 5437 | 209302\_at | POLR2H | Hs.432574 | polymerase (RNA) II (DNA directed) polypeptide H | 3 | 3q28 | Purine metabolism | 0.17 | 0 |
| Pyrimidine metabolism |
| RNA polymerase |
| 2707 | 215243\_s\_at | GJB3 | Hs.522561 | gap junction protein, beta 3, 31kDa (connexin 31) | 1 | 1p34 | Cell Communication | 0.17 | 0.23 |
| 26152 | 216692\_at | ZNF337 | Hs.213735 | zinc finger protein 337 | 20 | 20p11.21 | NA | 0.17 | 0 |
| Hs.633621 |
|  | 217107\_at | NA | NA | NA | NA | NA | NA | 0.17 | 0.33 |
| 382 | 203311\_s\_at | ARF6 | Hs.525330 | ADP-ribosylation factor 6 | 14 | 14q21.3 | Cholera - Infection | 0.17 | 0.23 |
| 55101 | 218038\_at | FLJ10241 | Hs.351099 | NA | 19 | 19q13.2 | NA | 0.17 | 0.27 |
| 58517 | 212030\_at | RBM25 | Hs.531106 | RNA binding motif protein 25 | 14 | 14q24.3 | NA | 0.17 | 0.33 |
| 51196 | 205112\_at | PLCE1 | Hs.144492 | phospholipase C, epsilon 1 | 10 | 10q23 | Inositol phosphate metabolism | 0.17 | 0.37 |
| Calcium signaling pathway |
| Phosphatidylinositol signaling system |
| 516 | 208972\_s\_at | ATP5G1 | Hs.80986 | ATP synthase, H+ transporting, mitochondrial F0 complex, subunit C1 (subunit 9) | 17 | 17q21.32 | Oxidative phosphorylation | 0.17 | 0.33 |
| ATP synthesis |
| 2999 | 210321\_at | GZMH | Hs.348264 | granzyme H (cathepsin G-like 2, protein h-CCPX) | 14 | 14q11.2 | NA | 0.17 | 0.17 |
| 3655 | 201656\_at | ITGA6 | Hs.133397 | integrin, alpha 6 | 2 | 2q31.1 | Cell Communication | 0.17 | 0.23 |
| Focal adhesion |
| ECM-receptor interaction |
| Cell adhesion molecules (CAMs) |
| Hematopoietic cell lineage |
| Regulation of actin cytoskeleton |
| 2961 | 202680\_at | GTF2E2 | Hs.77100 | general transcription factor IIE, polypeptide 2, beta 34kDa | 8 | 8p21-p12 | Basal transcription factors | 0.17 | 0.13 |
| 7277 | 212242\_at | TUBA1 | Hs.75318 | tubulin, alpha 1 | 2 | 2q35 | Gap junction | 0.17 | 0.06 |
| Pathogenic Escherichia coli infection - EHEC |
| Pathogenic Escherichia coli infection - EPEC |
| 116496 | 217967\_s\_at | C1orf24 | Hs.518662 | chromosome 1 open reading frame 24 | 1 | 1q25 | NA | 0.17 | 0 |
| 9177 | 221084\_at | HTR3B | Hs.241377 | 5-hydroxytryptamine (serotonin) receptor 3B | 11 | 11q23.1 | NA | 0.17 | 0.13 |
| 55129 | 218910\_at | TMEM16K | Hs.17949 | transmembrane protein 16K | 3 | 3p22.1-p21.33 | NA | 0.17 | 0.17 |
| 27324 | 214774\_x\_at | TNRC9 | Hs.460789 | trinucleotide repeat containing 9 | 16 | 16q12.1 | NA | 0.17 | 0.3 |
| 374655 | 37590\_g\_at | ZNF710 | Hs.459311 | zinc finger protein 710 | 15 | 15q26.1 | NA | 0.17 | 0.17 |
| 9776 | 209021\_x\_at | KIAA0652 | Hs.410092 | KIAA0652 | 11 | 11p11.2 | NA | 0.17 | 0.33 |
| 23001 | 212602\_at | WDFY3 | Hs.480116 | WD repeat and FYVE domain containing 3 | 4 | 4q21.23 | NA | 0.17 | 0.44 |
| 80215 | 220918\_at | C21orf96 | Hs.149261 | chromosome 21 open reading frame 96 | 21 | 21q22.12 | NA | 0.17 | 0.2 |
| 925 | 205758\_at | CD8A | Hs.85258 | CD8a molecule | 2 | 2p12 | Cell adhesion molecules (CAMs) | 0.17 | 0.44 |
| Antigen processing and presentation |
| Hematopoietic cell lineage |
| T cell receptor signaling pathway |
| 1500 | 208407\_s\_at | CTNND1 | Hs.166011 | catenin (cadherin-associated protein), delta 1 | 11 | 11q11 | Adherens junction | 0.17 | 0.23 |
| Leukocyte transendothelial migration |
| 64110 | 218176\_at | MAGEF1 | Hs.306123 | melanoma antigen family F, 1 | 3 | 3q13 | NA | 0.17 | 0.23 |
| 10099 | 200973\_s\_at | TSPAN3 | Hs.5062 | tetraspanin 3 | 15 | 15q24.3 | NA | 0.17 | 0.17 |
| 481 | 201242\_s\_at | ATP1B1 | Hs.291196 | ATPase, Na+/K+ transporting, beta 1 polypeptide | 1 | 1q24 | NA | 0.17 | 0.37 |
| 63940 | 214847\_s\_at | GPSM3 | Hs.520046 | G-protein signalling modulator 3 (AGS3-like, C. elegans) | 6 | 6p21.3 | NA | 0.17 | 0.4 |
| 1901 | 204642\_at | EDG1 | Hs.154210 | endothelial differentiation, sphingolipid G-protein-coupled receptor, 1 | 1 | 1p21 | Neuroactive ligand-receptor interaction | 0.17 | 0.33 |
| 5793 | 204944\_at | PTPRG | Hs.148340 | protein tyrosine phosphatase, receptor type, G | 3 | 3p21-p14 | NA | 0.17 | 0.06 |
| 8337 | 218280\_x\_at | HIST2H2AA3 | Hs.530461 | histone 2, H2aa3 | 1 | 1q21.2 | NA | 0.17 | 0.1 |
| 7280 | 204141\_at | TUBB2A | Hs.300701 | tubulin, beta 2A | 6 | 6p25 | Gap junction | 0.17 | 0.31 |
| Pathogenic Escherichia coli infection - EHEC |
| Pathogenic Escherichia coli infection - EPEC |
| 58528 | 221523\_s\_at | RRAGD | Hs.485938 | Ras-related GTP binding D | 6 | 6q15-q16 | NA | 0.17 | 0.27 |
| 30844 | 209536\_s\_at | EHD4 | Hs.143703 | EH-domain containing 4 | 15 | 15q11.1 | NA | 0.17 | 0.2 |
| 481 | 201243\_s\_at | ATP1B1 | Hs.291196 | ATPase, Na+/K+ transporting, beta 1 polypeptide | 1 | 1q24 | NA | 0.17 | 0.27 |
| 7056 | 203888\_at | THBD | Hs.2030 | thrombomodulin | 20 | 20p12-cen | Complement and coagulation cascades | 0.17 | 0.19 |
| 3556 | 205227\_at | IL1RAP | Hs.478673 | interleukin 1 receptor accessory protein | 3 | 3q28 | Cytokine-cytokine receptor interaction | 0.17 | 0.27 |
| Apoptosis |
| 22800 | 212589\_at | RRAS2 | Hs.502004 | related RAS viral (r-ras) oncogene homolog 2 | 11 | 11p15.2 | MAPK signaling pathway | 0.17 | 0.33 |
| Axon guidance |
| Tight junction |
| Regulation of actin cytoskeleton |
| 4722 | 201740\_at | NDUFS3 | Hs.502528 | NADH dehydrogenase (ubiquinone) Fe-S protein 3, 30kDa (NADH-coenzyme Q reductase) | 11 | 11p11.11 | Oxidative phosphorylation | 0.17 | 0.31 |
| 27240 | 205484\_at | SIT1 | Hs.88012 | signaling threshold regulating transmembrane adaptor 1 | 9 | 9p13-p12 | NA | 0.17 | 0.33 |
| 10670 | 201628\_s\_at | RRAGA | Hs.432330 | Ras-related GTP binding A | 9 | 9p22.1 | NA | 0.17 | 0.17 |
| 9249 | 202481\_at | DHRS3 | Hs.289347 | dehydrogenase/reductase (SDR family) member 3 | 1 | 1p36.1 | gamma-Hexachlorocyclohexane degradation | 0.17 | 0.3 |
| Bisphenol A degradation |
| 1- and 2-Methylnaphthalene degradation |
| Benzoate degradation via CoA ligation |
| Ethylbenzene degradation |
| Limonene and pinene degradation |
| 4791 | 209636\_at | NFKB2 | Hs.73090 | nuclear factor of kappa light polypeptide gene enhancer in B-cells 2 (p49/p100) | 10 | 10q24 | MAPK signaling pathway | 0.17 | 0.1 |
| Apoptosis |
| Toll-like receptor signaling pathway |
| T cell receptor signaling pathway |
| B cell receptor signaling pathway |
| Adipocytokine signaling pathway |
| Epithelial cell signaling in Helicobacter pylori infection |
| 55224 | 219268\_at | ETNK2 | Hs.497469 | ethanolamine kinase 2 | 1 | 1q32.1 | NA | 0.17 | 0.2 |
| 9402 | 208406\_s\_at | GRAP2 | Hs.517499 | GRB2-related adaptor protein 2 | 22 | 22q13.2 | T cell receptor signaling pathway | 0.17 | 0.23 |
| 23023 | 213349\_at | TMCC1 | Hs.477547 | transmembrane and coiled-coil domain family 1 | 3 | 3q21.3 | NA | 0.17 | 0.2 |
| 55657 | 220661\_s\_at | ZNF692 | Hs.377705 | zinc finger protein 692 | 1 | 1q44 | NA | 0.17 | 0.13 |
| 8728 | 209765\_at | ADAM19 | Hs.483944 | ADAM metallopeptidase domain 19 (meltrin beta) | 5 | 5q32-q33 | NA | 0.17 | 0.1 |
| 3915 | 200771\_at | LAMC1 | Hs.497039 | laminin, gamma 1 (formerly LAMB2) | 1 | 1q31 | Cell Communication | 0.17 | 0.26 |
| Focal adhesion |
| ECM-receptor interaction |
| Prion disease |
| 5600 | 206040\_s\_at | MAPK11 | Hs.57732 | mitogen-activated protein kinase 11 | 22 | 22q13.33 | MAPK signaling pathway | 0.17 | 0.31 |
| VEGF signaling pathway |
| Toll-like receptor signaling pathway |
| Fc epsilon RI signaling pathway |
| Leukocyte transendothelial migration |
| GnRH signaling pathway |
| Epithelial cell signaling in Helicobacter pylori infection |
| 4779 | 214179\_s\_at | NFE2L1 | Hs.514284 | nuclear factor (erythroid-derived 2)-like 1 | 17 | 17q21.3 | NA | 0.17 | 0.33 |
|  |  |  |  |  |  |  |  |  |  |
